# Molecular Basis of Class A β-Lactamase Inhibition by Relebactam

**DOI:** 10.1128/AAC.00564-19

**Published:** 2019-09-23

**Authors:** Catherine L. Tooke, Philip Hinchliffe, Pauline A. Lang, Adrian J. Mulholland, Jürgen Brem, Christopher J. Schofield, James Spencer

**Affiliations:** a School of Cellular and Molecular Medicine, Biomedical Sciences Building, University of Bristol, Bristol, United Kingdom; b Department of Chemistry, University of Oxford, Oxford, United Kingdom; c Centre for Computational Chemistry, School of Chemistry, University of Bristol, Bristol, United Kingdom

**Keywords:** antibiotic resistance, avibactam, diazabicyclooctane, relebactam, serine β-lactamase inhibitors

## Abstract

β-Lactamase production is the major β-lactam resistance mechanism in Gram-negative bacteria. β-Lactamase inhibitors (BLIs) efficacious against serine β-lactamase (SBL) producers, especially strains carrying the widely disseminated class A enzymes, are required. Relebactam, a diazabicyclooctane (DBO) BLI, is in phase 3 clinical trials in combination with imipenem for the treatment of infections by multidrug-resistant Enterobacteriaceae. We show that relebactam inhibits five clinically important class A SBLs (despite their differing spectra of activity), representing both chromosomal and plasmid-borne enzymes, i.e., the extended-spectrum β-lactamases L2 (inhibition constant 3 μM) and CTX-M-15 (21 μM) and the carbapenemases KPC-2, -3, and -4 (1 to 5 μM). Against purified class A SBLs, relebactam is an inferior inhibitor compared with the clinically approved DBO avibactam (9- to 120-fold differences in half maximal inhibitory concentration [IC_50_]). MIC assays indicate relebactam potentiates β-lactam (imipenem) activity against KPC-producing Klebsiella pneumoniae, with similar potency to avibactam (with ceftazidime). Relebactam is less effective than avibactam in combination with aztreonam against Stenotrophomonas maltophilia K279a. X-ray crystal structures of relebactam bound to CTX-M-15, L2, KPC-2, KPC-3, and KPC-4 reveal its C2-linked piperidine ring can sterically clash with Asn104 (CTX-M-15) or His/Trp105 (L2 and KPCs), rationalizing its poorer inhibition activity than that of avibactam, which has a smaller C2 carboxyamide group. Mass spectrometry and crystallographic data show slow, pH-dependent relebactam desulfation by KPC-2, -3, and -4. This comprehensive comparison of relebactam binding across five clinically important class A SBLs will inform the design of future DBOs, with the aim of improving clinical efficacy of BLI–β-lactam combinations.

## Abstract (precis)

β-Lactamase production is the major β-lactam resistance mechanism in Gram-negative bacteria. β-Lactamase inhibitors (BLIs) efficacious against serine β-lactamase (SBL) producers, especially strains carrying the widely disseminated class A enzymes, are required. Relebactam, a diazabicyclooctane (DBO) BLI, is in phase 3 clinical trials in combination with imipenem for the treatment of infections by multidrug-resistant Enterobacteriaceae.

## INTRODUCTION

A major determinant of drug resistance among Gram-negative pathogens is the production of β-lactamases, a large enzyme family whose members collectively hydrolyze all β-lactam antibiotics, including cephalosporins and last-resort carbapenems. β-Lactamases are divided into four main classes (A to D) based upon their sequences (1); classes A, C, and D are serine-β-lactamases (SBLs), while class B is the zinc-dependent metallo-β-lactamases (MBLs). SBLs hydrolyze antibiotics via formation of a hydrolytically labile acyl enzyme intermediate, while MBL catalysis proceeds without a covalent intermediate (2).

Of particular clinical importance is the widely disseminated class A SBLs, including the mobile, plasmid-encoded extended-spectrum CTX-M and carbapenem-hydrolyzing KPC families (both produced in opportunistic Gram-negative bacteria, such as Klebsiella pneumoniae, Pseudomonas aeruginosa, and Escherichia coli [3, 4]), as well as chromosomally encoded L2 from Stenotrophomonas maltophilia (a lung colonist of cystic fibrosis patients [5, 6]). CTX-M and KPC production significantly threatens current antimicrobial chemotherapy (7, 8). CTX-M-15 is one of the most important members of the CTX-M extended-spectrum β-lactamase (ESBL) family, with a wide spectrum of catalytic activity (3, 8). Of the KPC carbapenemases, KPC-2, KPC-3, and KPC-4 are the most prevalent in resistant Enterobacteriaceae, differing from KPC-2 by single- or double-point substitutions at amino acid positions 104, 240, and 274 (KPC-3 H274Y; KPC-4 P104R/V240G) that change β-lactam specificity, especially toward the oxyimino-cephalosporin ceftazidime (7). L2 (an SBL) is one of two intrinsic β-lactamases, with L1 (an MBL), which together provide resistance to all β-lactams, making S. maltophilia one of the most extensively drug-resistant pathogens in the clinic and one of the most difficult to treat (5).

Three classical β-lactam-based β-lactamase inhibitors (BLIs), i.e., clavulanic acid (9), sulbactam, and tazobactam, are used extensively to potentiate β-lactam activity (10). Inhibition is achieved through the formation of (an) irreversible, covalent adduct(s) with the catalytic serine of SBLs. These inhibitors have clinically useful (10) potency against class A SBLs but not typically against enzymes of classes C or D. Since their introduction, some class A SBLs have accumulated mutations resulting in inhibitor resistance (11), while enzymes such as KPC show reduced susceptibility to inhibition (12). These observations highlight the need for novel BLIs effective against a wider range of β-lactamases.

**FIG 1:**
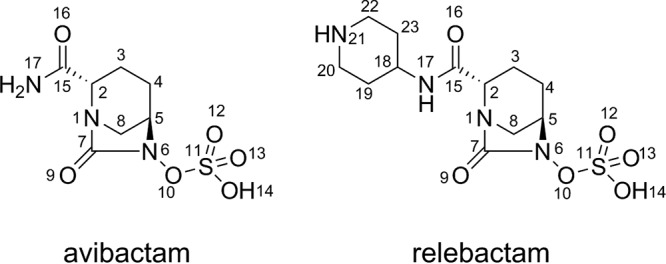
Structures of the diazabicyclooctanes (DBOs) avibactam and relebactam.

The diazabicyclooctanes (DBOs), including avibactam (13), relebactam (14), and others (15–17), are a new BLI class with improved activity against a wider range of SBL targets than classic BLI scaffolds. Avibactam and relebactam contain the same bicyclic DBO core and differ in their side chains; relebactam contains an additional piperidine ring at C2 (Fig. 1). DBOs inhibit SBLs through covalent formation of a carbamyl ester to the active-site serine concomitant with DBO ring opening. In contrast to clavulanic acid, binding is reversible, with decarbamylation and recyclization observed in CTX-M-15 (18, 19), TEM-1 (13), and KPC-2 (13), as indicated by using acyl exchange between two serine-β-lactamases. Mass spectrometry of avibactam binding to KPC-2 shows slow hydrolysis over 24 h, likely following desulfation of the substrate (20). However, under similar conditions, relebactam desulfation by KPC-2 was not observed (16), with molecular dynamics suggesting this enhanced relebactam stability results from repositioning of active site water molecules (21, 22).

In 2015, a ceftazidime-avibactam combination (Avycaz/Zavicefa) was approved for the treatment of complicated urinary tract and abdominal infections. This combination expands ceftazidime activity to encompass Gram-negative bacteria producing ESBLs and KPCs. More recently, an imipenem-relebactam combination is in phase 3 clinical trials, restoring the imipenem sensitivity of some resistant K. pneumoniae and P. aeruginosa (22). However, as with classical BLIs, avibactam resistance is emerging due to mutations/deletions in the β-lactamase target (11, 23); several laboratory-generated mutants have provided insight into the potential mechanisms for avibactam and likely relebactam resistance (24).

Structural investigations of relebactam are limited to the class C β-lactamase AmpC from Pseudomonas aeruginosa at 1.9-Å resolution (PDB identifier 4NK3 [14]). Here, we investigate the structural basis of relebactam inhibition of 5 class A β-lactamases, correlating the results with differences in hydrolytic performance. The ESBLs CTX-M-15 (3) and L2 (25) confer resistance to penicillins, first-, second-, and third-generation cephalosporins, and the monobactam aztreonam but are unable to hydrolyze carbapenems, while the hydrolytic capabilities of the KPC carbapenemases (KPC-2, KPC-3, and KPC-4) extend to the potent “last resort” carbapenems (7, 26). We also provide biochemical and microbiological data to investigate the differences in DBO inhibition across these enzyme families that will inform the design of future inhibitor generations.

## RESULTS AND DISCUSSION

### Relebactam restores imipenem susceptibility of KPC-producing K. pneumoniae but is less effective against S. maltophilia.

The imipenem:relebactam combination (Merck) is currently undergoing phase 3 clinical trials, in particular for the treatment of serious infections caused by carbapenem-resistant *Enterobacteriaceae* (ClinicalTrials.gov identifier NCT02452047). *In vitro* studies have shown that both the ceftazidime:avibactam and imipenem:relebactam combinations are effective against clinical *Enterobacteriacae* isolates, producing either KPC-2 or KPC-3 (27, 28). However, other KPC variants vary more profoundly in their activities against specific β-lactams (7), while relebactam activity against the nonfermenting species S. maltophilia is little explored. Accordingly, we compared susceptibilities of recombinant K. pneumoniae Ecl8 (29) producing the three most prevalent KPC variants, namely, KPC-2, KPC-3, or KPC-4, to determine the efficacy of relebactam combinations against SBL variants with different β-lactam-hydrolyzing capabilities and extended these experiments to include the clinical S. maltophilia K279a isolate. S. maltophilia causes myriad multidrug-resistant infections, often in immunocompromised patients, and is, therefore, a particularly challenging target for antimicrobial therapy (6). Recently, an avibactam:aztreonam combination proved successful in the treatment of several S. maltophilia strains (30); we investigate whether this activity is reflected with a relebactam:aztreonam combination.

β-Lactam MICs for KPC variants expressed in K. pneumoniae Ecl8 range from 16 mg liter^−1^ to 128 mg liter^−1^ for ceftazidime and 0.5 mg liter^−1^ (KPC-4) to 16 mg liter^−1^ or 64 mg liter^−1^ for imipenem (Table 1). This range of MICs is reflected in previously determined *k*_cat_ values (7) and relative MICs and hydrolysis rates (31) for KPC variants with both substrates. Despite these differences, imipenem MICs are lowered to ≤0.5 mg liter^−1^ in all KPC producers in the presence of 4 mg liter^−1^ relebactam (Table 1), similar to the efficacy of a ceftazidime:avibactam combination. Both combinations can, therefore, be successful in treating strains producing the range of KPCs (with variable β-lactam-hydrolyzing capabilities) in clinical, pathogenic *Enterobacteriaceae*. In contrast, the S. maltophilia K279a clinical isolate (which produces both L1 [an MBL] and L2 [an SBL]) is resistant to both imipenem and the imipenem:relebactam combination (Table 1). We ascribe this to the presence of the L1 MBL that is able to hydrolyze imipenem and is not inhibited by DBOs (25). However, we, and others (30), have recently demonstrated that several strains of S. maltophilia (including K279a) can be inhibited with the monobactam aztreonam (which is not hydrolyzed by L1) combined with a nonclassical BLI (25, 30). Indeed, an avibactam:aztreonam combination has been shown to be effective in treating a S. maltophilia infection in the clinic (30). Accordingly, we investigated the combination of aztreonam with relebactam against S. maltophilia K279a. The addition of relebactam lowers aztreonam MICs from 256 mg liter^−1^ to 8 mg liter^−1^ (Table 1), but this compares unfavorably with avibactam, for which aztreonam MICs were lowered to 2 mg liter^−1^ (25). Thus, while effective against KPC-producing K. pneumoniae, compared with avibactam, relebactam combinations (in particular with aztreonam) appear to be less effective against S. maltophilia.

**TABLE 1:** MICs of β-lactams against S. maltophilia or K. pneumoniae in the presence of β-lactamase inhibitors

Strain or plasmid	MIC (μg/ml) of β-lactams by inhibitor^*a*^					
	Ceftazidime		Imipenem		Aztreonam	
	−	+AVI (4 mg/liter)	−	+REL (4 mg/liter)	−	+REL (4 mg/liter)
S. maltophilia K279a	16	8	128	64	>256	8
Ecl8 pUBYT	<0.125		<0.0625			
Ecl8 pUBYT KPC-2	16	0.125	64	0.5		
Ecl8 pUBYT KPC-3	128	<0.125	16	<0.125		
Ecl8 pUBYT KPC-4	128	1	0.5	0.125		

a AVI, avibactam; REL, relebactam; −, no inhibitor present.

### Relebactam is a potent inhibitor of class A β-lactamases *in vitro*.

**FIG 2:**
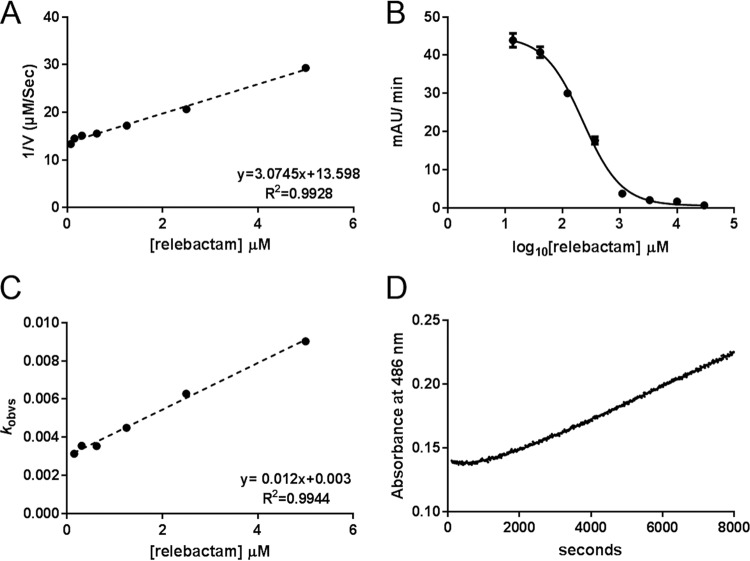
Kinetic characterization of relebactam inhibition of KPC-2. (A) Dixon plot of reciprocals of initial nitrocefin hydrolysis rates (1/*V*) by enzyme:relebactam mixtures plotted against relebactam concentration. The apparent inhibition constant *K*_iapp_ is obtained from the slope of the fitted straight line. (B) Initial rates of nitrocefin hydrolysis (absorbance units/min) after 10-minute incubation with relebactam, plotted against log_10_ [relebactam]. Fitted curve is used to derive IC_50_ according to equation 1. (C) Plot of *k*_obs_ (pseudo-first-order rate constant for inactivation) against relebactam concentration. The apparent second-order rate constant for the onset of carbamylation *k*_2_/*K* is obtained from the slope of the fitted straight line. (D) Progress curve representing recovery of nitrocefin hydrolysis following 10-minute preincubation of enzyme (1 μM) with 17.5 μM relebactam, diluted to a final concentration of 50 nM enzyme. The rate of recovery of free enzyme, *k*_off_, is obtained from the fitted line shown according to equation 8. Data points shown are means of three replicate runs.

Prior kinetic characterization (21) reveals relebactam to be a potent, micromolar, competitive inhibitor of KPC-2. We characterized the inhibition by relebactam, determining values for half maximal inhibitory concentration (IC_50_), the apparent dissociation constant for the inhibitory complex (*K*_iapp_) as determined from Dixon plots (32), the apparent second-order rate constant for the onset of carbamylation by relebactam (*k*_2_/*K*), and rate of recovery of free enzyme (*k*_off_), of five class A β-lactamases, the ESBLs CTX-M-15 and L2, and the carbapenemases KPC-2, KPC-3, and KPC-4 (Fig. 2 and Fig. S1 to S4 in the supplemental material) and compared these values with those for avibactam. The IC_50_ values (Table 2) determined after a 10-minute preincubation time with the inhibitor indicate that avibactam potently inhibits CTX-M-15 (3.4 nM), with potency decreasing 3-fold for KPC-2 (IC_50_, 10 nM) and KPC-4 (9.3 nM), 5-fold for L2 (15 nM), and 8-fold for KPC-3 (29 nM). Relebactam is substantially less potent than avibactam for each enzyme by 9-fold (KPC-3), 22-fold (KPC-2), 31-fold (L2), 98-fold (KPC-4), or 119-fold (CTX-M-15). The trends in avibactam and relebactam inhibition across these 5 enzymes are not consistent, for example CTX-M-15 is the most sensitive to avibactam (lowest IC_50_) but the least sensitive to relebactam (highest IC_50_) (Table 2). Importantly, our data indicate that for the tested enzymes, relebactam is consistently a substantially inferior inhibitor compared with avibactam (IC_50_, 230 to 910 nM; compared to 3.4 to 29 nM) (Table 2). Furthermore, the >30-fold increase in IC_50_ between avibactam and relebactam for L2 likely explains the difference in effectiveness of the respective aztreonam combinations against S. maltophilia K279a. However, against KPC-expressing K. pneumoniae, relebactam combinations are as effective as those with avibactam, suggesting that, in organisms more permeable than S. maltophilia, differences in *in vitro* potency between the two DBOs do not translate into effects on MIC for their respective combinations.

**TABLE 2:** IC_50_ values for DBO inhibitors against class A β-lactamases^*a*^

Protein	IC_50_ values (nM) by inhibitor	
	Avibactam	Relebactam
CTX-M-15	3.4 (0.02)	400 (0.04)
L2	15 (0.02)	470 (0.03)
KPC-2	10 (0.05)	230 (0.03)
KPC-3	29 (0.03)	260 (0.05)
KPC-4	9.3 (0.08)	910 (0.03)

a Standard errors of log IC_50_ values are shown in parentheses.

A more detailed investigation of the time dependence of relebactam inhibition (Table 3) showed that *K*_iapp_ values, derived from Dixon plots based upon progress curves of initial rates of nitrocefin hydrolysis for enzyme:relebactam mixtures that had not been preincubated, generally reflect the IC_50_, with the exception of CTX-M-15, which has a relatively high *K*_iapp_ value of 21 μM. We consider this high value to reflect the atypically slow carbamylation of the enzyme by relebactam, with a second-order rate constant for carbamylation *k*_2_/*K* of 540 M^−1^ s^−1^ (Table 3). These values are also consistent with others’ recent reports of *K*_iapp_ values for relebactam inhibition of KPC-2 (2.3 μM) (21) and the *Pseudomonas*-derived cephalosporinase-3 (PDC-3) enzyme (3.4 μM) (33).

**TABLE 3:** Kinetic parameters for relebactam inhibition^*a*^

Extended-spectrum β-lactamase	Values by kinetic parameter:			
	*K*_iapp_ (μM)	*k*_2_/*K* (M^−1^ s^−1^)	*k*_off_ (s^−1^)	*t*_1/2_ (min)
L2	2.7 (0.7)	4,000 (620)	0.00055 (0.000021)	21
CTX-M-15	21.0 (1.0)	540 (19)	0.00038 (0.000053)	30
KPC-2	1.2 (0.05)	4,500 (220)	0.00087 (0.000032)	13
KPC-3	1.5 (0.05)	2,100 (140)	0.00020 (0.000035)	58
KPC-4	4.8 (0.7)	1,100 (190)	0.00019 (0.000038)	61

a Errors in parentheses represent standard deviation (*K*_iapp_ and *k*_2_/*K*) or standard error from fits of (*k*_off_) from measurements carried out in triplicate.

Of the three KPC variants studied, KPC-4 has the highest apparent inhibition constant for relebactam (*K*_iapp_, 4.8 μM; compared to essentially identical *K*_iapp_ values for KPC-2 [1.2 μM] and KPC-3 [1.5 μM]). Differences between the three KPC variants are noticeable in their carbamylation rate constants *k*_2_/*K*, with values for KPC-2 (4,500 ± 220 M^−1^ s^−1^) noticeably faster than for KPC-3 (2,100 ± 140 M^−1^ s^−1^) or KPC-4 (1,100 ± 190 M^−1^ s^−1^). The effect of this is, however, ameliorated by a reduction of ∼4.5-fold in off-rate for both of these variants compared with KPC-2. For L2 (*K*_iapp_, 2.7 μM), both the second-order carbamylation rate constant (4,000 ± 620 M^−1^ s^−1^) and off-rate (0.00055 ± 0.00021 s^−1^) are relatively high. Overall differences in carbamylation rate across the five SBLs tested span almost 1 order of magnitude, while those in off-rate extend to <5-fold (Table 3).

### The structural basis for relebactam inhibition of class A β-lactamases.

To investigate the molecular basis for relebactam inhibition of class A β-lactamases and to identify structural explanations for differences in potency, we soaked crystals of CTX-M-15, L2, KPC-2, KPC-3, and KPC-4 with relebactam. For comparison, we also describe the crystal structures of native KPC-3 and KPC-4 at 1.22- and 1.42-Å resolution (see Table S3 in the supplemental material; see Fig. S5A and S5B in the supplemental material). A comparison of these structures confirms that, compared to KPC-2, the H274Y and P104R/V240G substitutions do not result in large global conformational changes (Fig. S5C; Table S5) or induce significant perturbations of the active site (Fig. S5D).

**FIG 3:**
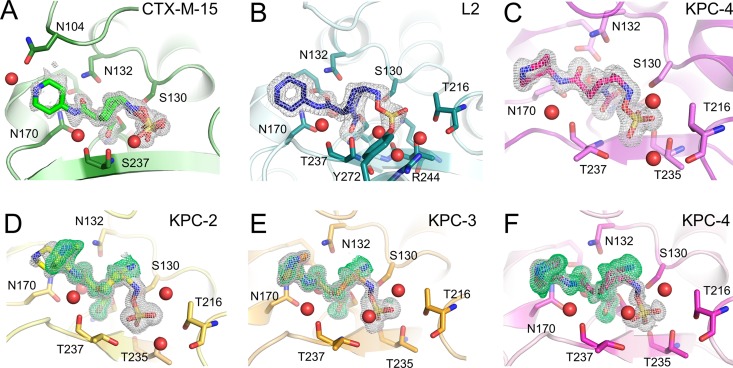
Electron density maps showing relebactam bound to the active sites of class A β-lactamases. Unbiased omit *F*_o_-*F*_c_ electron density maps were calculated with the ligand removed and are shown contoured at 3σ. Gray density is calculated after removal of “intact” relebactam, green density after removal of desulfated relebactam. (A) CTX-M-15 (green, 16-hour soak), (B) L2 (teal, 16 hours), (C) KPC-4 (pink, 1 hour), (D) KPC-2 (yellow, 16 hours), (E) KPC-3 (orange, 16 hours), and (F) KPC-4 (16 hours).

Relebactam cocomplex structures were obtained after 16-h soaks for CTX-M-15 (1.12 Å, *P*2_1_2_1_2_1_), L2 (1.78 Å, *P*2_1_2_1_2_1_), KPC-2 (1.04 Å, *P*2_1_2_1_2), KPC-3 (1.06 Å, *P*2_1_2_1_2), and KPC-4 (1.04 Å, *P*2_1_2_1_2) (Table S3). We also obtained a crystal structure for a KPC-4 relebactam complex from data collected after a 1-hour soak. For all of these complex structures, there was clear *F*_o_-*F*_c_ difference density in the active site into which relebactam could be modelled (Fig. 3), with ligand real-space correlation coefficients (RSCCs) all greater than 0.93 (see Table S4 in the supplemental material). This combination of high resolution and strong difference density enabled us to model the bound inhibitor with a high degree of confidence and enabled us to identify alternative ligand conformations and structures where these were present. For L2, electron density consistent with a single conformation of relebactam (refined at full occupancy) was observed in one of the two molecules in the asymmetric unit (chain B). Consistent with previous observations for other potent L2 inhibitors in this crystal form, we observe a noncovalently bound molecule from the crystallization solution (d-serine) in the chain A active site (25). In CTX-M-15, relebactam could be refined in two conformations, with occupancies of 0.49 and 0.51. In the KPC variants, structures obtained from diffraction data sets collected after exposing crystals to relebactam for 16 hours were observed to contain both intact and desulfated (i.e., in the imine form) relebactam in the active site, at variable occupancies. For comparison, a KPC-4 structure obtained after the crystal was exposed to inhibitor for just 1 h revealed only intact relebactam covalently bound in the active site.

### Relebactam interactions with class A β-lactamases.

**FIG 4:**
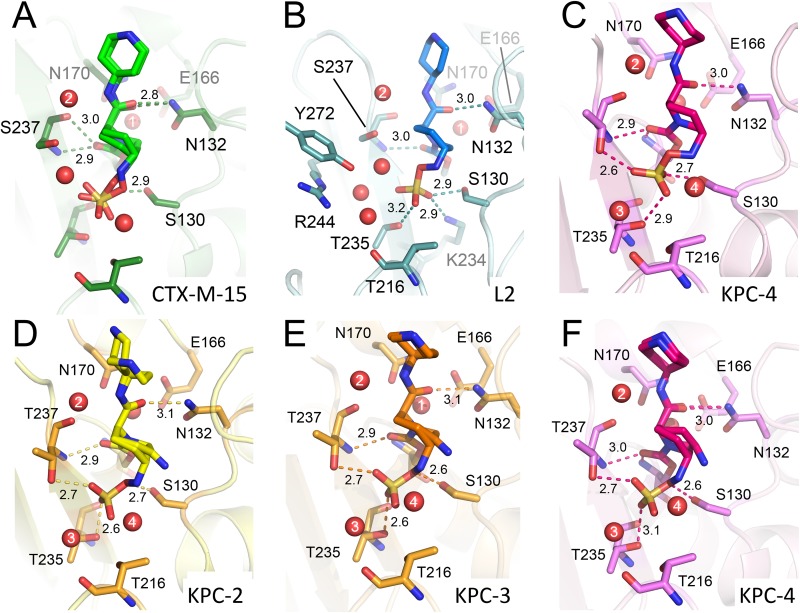
Interactions of relebactam with active sites of class A β-lactamases. Close-up views of relebactam bound in the active sites of class A β-lactamases (colored as in Fig. 3). Hydrogen bonding interactions of relebactam with the protein main chain are shown as dashes with distances in Å. Water molecules are shown as red spheres; those that make conserved interactions are numbered. (A) CTX-M-15 (16-hour soak) showing relebactam bound in two conformations, (B) L2 (16-hour soak), and (C) KPC-4 (1-hour soak). Relebactam was modelled as both the imine (desulfated, acyl-98 as shown in Fig. 5A) and intact forms from 16-hour soaks of (D) KPC-2, (E) KPC-3, and (F) KPC-4 crystals.

The crystal structures of all five class A SBLs tested here reveal relebactam covalently attached to the nucleophilic Ser70 (Fig. 3 and 4; see Fig. S6 to S8 in the supplemental material). Binding causes no apparent global conformational changes (for comparison, RMSDs between the relevant structures are provided in Table S5) and no large changes in any of the active sites compared with those of the native, uncomplexed enzymes. Importantly, the positioning of the deacylating water (Wat1) is apparently little affected by relebactam binding (Fig. S6 to S8). As seen in previous DBO complex structures (16, 19, 25), relebactam binds as a ring-opened carbamoyl-enzyme complex (13, 14), whereby the six-membered ring adopts a chair conformation (Fig. 3 and 4; Fig. S6 to S8). The deacylating water, similarly positioned by Glu166 and Asn170 in all complexes, lies close to the C7 atom (see Fig. 1 for atom numbering) of relebactam (2.9 Å to 3.2 Å), and is apparently positioned for decarbamylation (Fig. S6 to S8, panels B and D). Differences we observe between enzymes in *k*_off_ (i.e., the decarbamylation rate of the acyl complex) are, therefore, probably at least not solely due to changes in the position of the deacylating water. A second active site water molecule (Wat2), and its interactions with residues 237 and the N17 atom of relebactam, is also conserved across the 5 enzymes (Fig. S6A and D, S7A and D, and S8A and D).

### Comparisons of DBO binding.

The efficacy of avibactam and extensive research into structure-activity relationships (SAR) has prompted the development of further generations of DBOs with modifications to the R1 side chain, including relebactam. Comparisons of relebactam binding with other KPC-2, L2, and CTX-M-15 DBO complexes reveal common modes of binding for the inhibitor core. The avibactam carboxyamide side chain in KPC-2 (PDB identifier 4ZBE) adopts a similar geometry to the O16 and N17 atoms of relebactam, with the only difference being the position of Wat2 (see Fig. S10A in the supplemental material). Despite this movement, Wat2 still hydrogen bonds to the N17 atom in both avibactam and relebactam complexes with KPC-2 (34). WCK-5107, also known as zidebactam, has a *K_i_* value against KPC-2 of 5-fold higher than avibactam and 2-fold higher than relebactam but only differs from relebactam by an additional amine at position 18. In a KPC-2 complex structure obtained after a 3-h soak (PDB identifier 6B1J) (16) (Fig. S10B), the piperidine ring of WCK-5107 binds closer (by approximately 3.0 Å) to the backbone oxygen of Cys238 than that of relebactam. The disulfide bond that this residue forms with Cys69 is known to be important to the hydrolytic activity, including carbapenemase activity, of KPC enzymes (35, 36).

In the cocrystal structure of the WCK-5107 complex (Fig. S10C), a desulfated, imine form of the DBO is modelled, binding similarly to the imine conformation of relebactam observed here. WCK-4234, the most potent DBO described by Papp-Wallace et al. (16), with cross-class activity against SBLs, binds to KPC-2 with its R1 side chain pointing toward Asn132, in contrast to the N17 of relebactam, which points in the opposite direction (Fig. S10D). As with other DBOs, the N6, O10, and sulfate moiety of WCK-4234 are all flexible and modelled in a range of different conformations compared with relebactam.

In both CTX-M-15 and L2, DBO binding modes are similar, with only a small rotation of the R1 carboxyamide when comparing avibactam and relebactam (Fig. S10E and S10F). In L2, this results in an additional water molecule, which is not present in the avibactam complex, that bridges N17 of relebactam (Wat2) with the Ser237 side chain oxygen. For each of the comparisons described above (Fig. S10), the sulfate moiety and attached O10 atom adopt subtly different conformations in each of the complexes, particularly compared with relebactam. This observation implies flexibility in binding for this region of the DBOs, although numerous factors may underline the differences, including the different resolution limits for each crystal structure, soaking times, or crystallization conditions, and in the case of L2, additional interacting water molecules in the active site.

### Hydrogen bonding of relebactam in class A active sites.

Relebactam is positioned to form hydrogen bonds with the oxyanion hole (Ser70 and Thr237 backbone amides), Asn132, and Ser130, residues that are all conserved (Fig. 4) in the five enzymes. In the L2 and KPC variants, the Thr216 backbone oxygen also partakes in hydrogen bond networks (via a water molecule, Wat3, in KPCs and Wat3-5 in L2) (Fig. 4) to the relebactam sulfate, an interaction absent in the CTX-M-15 complex. This is probably due to the flexible binding of the sulfate moiety, as described above. In addition, the L2 structure contains two active-site water molecules uniquely observed bridging relebactam and residues Tyr272, Arg244, Lys234, and Gly236 (Fig. S6). These additional interactions in L2 and the KPCs may contribute to the smaller *K_i_* values than CTX-M-15 (Table 3).

### Flexibility in residues 104 (CTX-M-15) and 105 (L2, KPCs) is important for relebactam binding.

Residues 104 and 105 lie at the entrance of the active site in all class A β-lactamases. Residue 105 has been investigated extensively in TEM-1 (37, 38), SME-1 (35), and KPC-2 (39) and is thought to have important roles in discriminating between and stabilizing bound substrates/intermediates during hydrolysis (see Fig. S9 in the supplemental material). In the structure of unliganded KPC-2 (PDB identifier 5UL8), Trp105 has a poorly defined electron density, suggestive of the presence of conformational flexibility (40). Indeed, this is also the case in our unliganded KPC-3 and KPC-4 structures, solved in the same space group (*P*2_1_2_1_2), where Trp105 is modelled in two conformations (Fig. S9). This movement has only been observed in KPC-2 crystal structures solved in the space groups *P*2_1_2_1_2 or *P*22_1_2_1_. In other KPC-2 crystal structures solved in different space groups, e.g., PDB identifiers 3DW0 (41) and 2OV5 (42), the Trp105-containing loop is stabilized by crystal contacts and Trp105 movement is not observed. In crystal structures of the hydrolysis products of cefotaxime and faropenem noncovalently bound to KPC-2 (40) (space group *P*22_1_2_1_), Trp105 is modelled in one conformation into clear electron density, revealing that substrate and/or product binding stabilizes the conformation of this residue. However, in the KPC-2:relebactam complex, both Trp105 and the relebactam piperidine ring are modelled in two conformations (Fig. 3D and 4D; Fig. S7A and S9D).

In KPC-3 and -4 relebactam complexes, Trp105 is modelled in one conformation, similar to one of the two conformations observed in KPC-2, albeit with high B-factors (30.95 and 33.19 compared with average protein B-factors of 13.61 and 13.24, respectively), suggesting there is still flexibility and movement of this residue. In this conformation, Trp105 faces the DBO core, with the nitrogen of the pyrrole ring ∼3.0 Å from the relebactam imine N6 (Fig. S9). This is concomitant with well-defined electron density for the relebactam piperidine ring (Fig. 3). Therefore, movement of Trp105 and binding of the piperidine ring appear linked, with the potential for steric clashes to occur if Trp105 was positioned to face the C2 side chain. While the flexibility of this residue may be allowing the KPC-2 active site to accommodate relebactam, the necessity of rearrangement to avoid these steric clashes likely contributes to the decrease in potency of relebactam compared with avibactam.

In the CTX-M-15:relebactam complex, unlike the other SBLs studied here, electron density, for both Asn104 and the relebactam piperidine ring is poorly defined. In crystal structures of wild-type CTX-M enzymes, Asn104 is well-defined by experimental electron density but is positioned to clash sterically with the expected orientation of the piperidine ring of bound relebactam (Fig. 3 and Fig. S9 in the supplemental material). Thus, relebactam binding appears to increase the conformational flexibility of CTX-M-15 Asn104 in order to escape such unfavorable interactions. We propose that the need to reposition Asn104 on relebactam binding contributes to the slower carbamylation rate and higher *K*_iapp_ values (Table 3) for CTX-M-15, compared with the other enzymes tested here.

In L2, two conformations of His105 are observed on relebactam binding, with one configuration the same as that observed in the L2:avibactam and native structures, which each contain a single His105 conformation. As in CTX-M-15, these movements are not observed in unliganded enzyme or in the avibactam-bound L2 structure (25). These energetically unfavorable clashes may indicate why relebactam is 30-fold worse than avibactam at inhibiting L2 (Table 2).

With these observations in mind, it is noteworthy that, of the DBO compounds tested to date, the compound with the shortest R1-group, WCK-4234, exhibits the greatest potency (*K_i_*) against KPC-2. This may be explained by comparisons of the crystal structures of KPC-2 complexed with WCK-4234 and relebactam (Fig. S10D); notably, the nitrile R-group of bound WCK-4234 points away from Trp105, whereas the relebactam (piperidine-containing) R-group adopts multiple conformations, of which some clash with Trp105.

### Crystal structures of SBL:relebactam complexes reflect two potential pathways for relebactam release.

**FIG 5:**
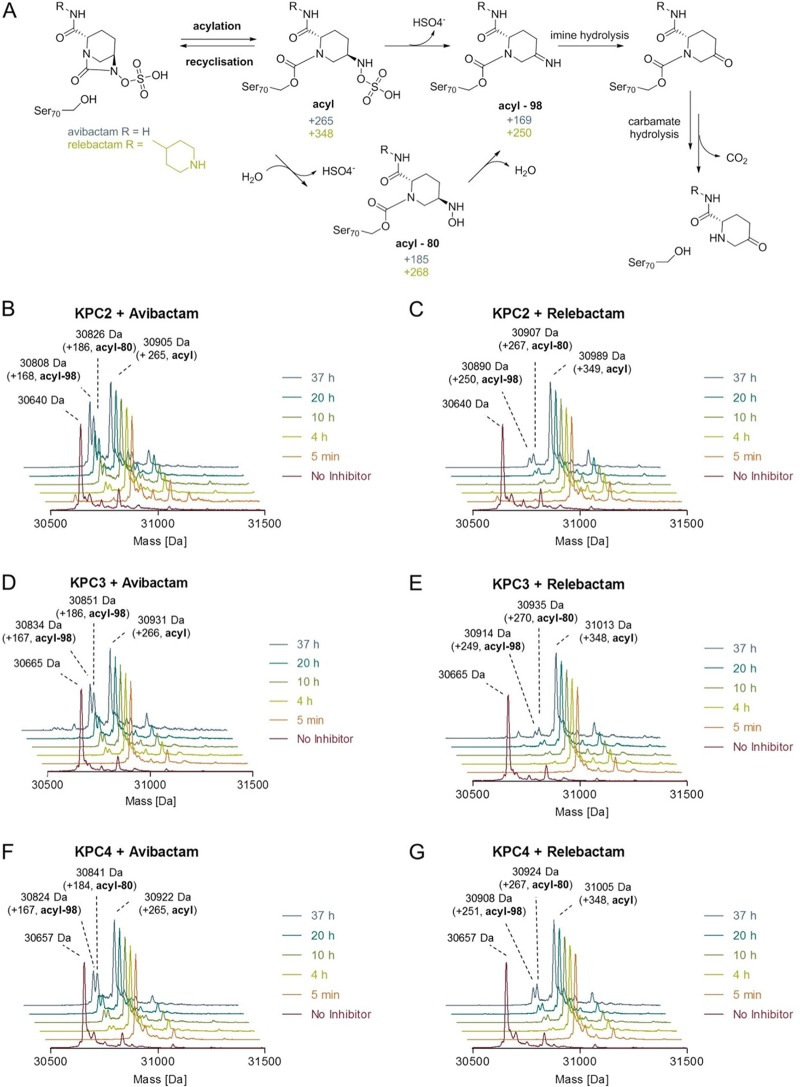
Time-dependent fragmentation of covalent avibactam and relebactam adducts. (A) Chemical structures of proposed intermediates upon carbamylation and fragmentation pathways. (B, C, D, E, F, G) Deconvoluted ESI-MS spectra showing covalent modifications of KPC variants (KPC-2, -3, and -4) by DBOs over time. Enzymes were incubated with 2 equivalents of avibactam or relebactam in 50 mM Tris-HCl (pH 7.5) at room temperature. Data are shown after maximum entropy deconvolution (MassHunter Qualitative Analysis software v.7 [Agilent Technologies]) over the mass range 1,200 to 2,000 Da.

Two pathways for avibactam release (Fig. 5) are postulated to occur, namely, decarbamylation after DBO recyclization or decarbamylation by direct hydrolysis after the loss of the inhibitor sulfate. First, Ehmann et al. observed, in experiments monitoring transfer of the acylating group between the class A enzymes TEM-1 and CTX-M-15, that decarbamylation occurs predominantly through regeneration of intact avibactam (i.e., recyclization) (13). Second, in complexes with KPC-2, the avibactam acyl-enzyme can slowly hydrolyze without recyclization, with the observation that only 10% of KPC-2 remains acylated after 24 hours of incubation, which is suggestive of a slow, hydrolytic mechanism (20). In time course experiments monitoring the stability of the KPC-2:avibactam acyl-enzyme, two new acyl-enzyme peaks were identified by MS, indicating losses of 79 and 98 Da, consistent with the loss of SO_4_^2−^(desulfation) with formation of either hydroxylamine or imine fragments (Fig. 5). It is thought that these fragmentations precede avibactam loss by hydrolysis and result in relief of inhibition as the released fragments are incapable of forming intact DBO by recyclization.

We, therefore, examined our various relebactam complex crystal structures with the aim of establishing their compatibility with these competing pathways for the loss of covalent attachment from the enzyme. In the CTX-M-15 complex, electron density indicates that bound relebactam is in two clear conformations, with occupancies of 0.51 and 0.49 (Fig. 3A). In one of these two conformations, the relebactam N6 atom interacts closely (2.9 Å) with Ser130, leaving N6 closer to the carbamoyl group than Wat1, and resembling the recyclization “primed” state previously reported for class A SBLs (12) (PDB identifier 4HBU) (Fig. S6F). Additionally, short hydrogen bond distances (2.9 Å) are observed between Lys73 and Ser130, similar to those found in the avibactam crystal structure (PDB identifier 4S2I). Lys73 has been proposed to act as a general base for Ser130 activation for avibactam recyclization (19); the crystal structure of CTX-M-15:relebactam presented here identifies that this is likely also the case for relebactam. This “recyclization primed” conformation for class A enzyme-bound DBOs has only been previously observed in the CTX-M-15:avibactam (19) and the KPC-2:WCK-4234 complexes (16) (Fig. S10). However, we also note the presence of a second conformation of relebactam in CTX-M-15 that closely resembles that found in avibactam complexes of other enzymes, with N6 positioned further from Ser130 (3.5 to 4.1 Å) and C7 (Fig. S6C and F, S7C and F, and S8C and F), i.e., not primed for recyclization (Fig. S6D, E, and F). The presence of these alternative conformations seems to have little impact on DBO off-rates, with previous studies determining off rates of 1.4 × 10^−4^ s^−1^ to 6.7 × 10^−4^ s^−1^ for avibactam from CTX-M-15 (20, 43, 44) and 4.5 × 10^−4^ s^−1^ ± 0.5 × 10^−4^ s^−1^ for WCK-4234 (16) from KPC-2, similar to the relebactam off-rates observed here (Table 3).

In the complex with L2, which contains intact (i.e., nondesulfated but ring-opened) relebactam in a single conformation, Lys73 and Ser130 were similarly close to one another (Fig. 4 and Fig. S6A, B, and C). Despite this proximity, Ser130 is ∼3.9 Å away from the N6 nitrogen, and so relebactam appears to still not be primed for recyclization in L2. However, in each of the three KPC complexes, in all of which relebactam was a modelled as a mixture of intact and desulfated forms, Lys73 is at least 0.4 Å more distant from Ser130 than is the case for either the CTX-M-15 (either conformation) or L2 structures. These increased distances, when considered with the postulated recyclization pathway that involves proton transfer from Ser130 to Lys73, in addition to the distance of at least 3.5 Å between Ser130 and the N6 of relebactam, suggest that recyclization is less favorable in the KPC complexes than in those formed with the other two enzymes.

Consistent with this possibility, in the KPC-2, KPC-3, and KPC-4 structures, inspection of electron density maps indicated the presence of desulfated relebactam, which could be modelled as the imine form of the inhibitor, with occupancies of 0.35, 0.33, and 0.65 for KPC-2, -3, and -4, respectively. The imine group points toward the flexible Trp105, but otherwise, the DBO core closely resembles intact relebactam.

It had previously been thought that relebactam complexes with class A SBLs did not undergo desulfation, with mass spectrometry experiments with KPC-2 suggesting that fragmentation was not occurring even after 24 hours of incubation (16, 21). However, our KPC complex structures provide crystallographic evidence supporting potential relebactam desulfation, at least *in crystallo*, after 16 hours of incubation. To further investigate the possibility of relebactam desulfation, liquid chromatography-electrospray ionization-mass spectrometry (LC-ESI MS) studies were carried out on the full-length (codons 25 to 293) proteins at a range of time points after exposure to both avibactam and relebactam (Fig. 5 and Table 4). All KPC variants tested manifested apparently complete carbamylation, without significant fragmentation, by both DBOs within 5 min, which is in agreement with the fast on-rates we observe kinetically. Initially, acquired spectra showed only adducts of +265 Da and +348 Da, respectively, indicating carbamylation of intact avibactam and relebactam, respectively. Over a period of 37 h, gradual fragmentation of the complexes to adducts with masses decreased by 80 Da and 98 Da (forming the hydroxylamine and imine species, respectively), compared with the initial acyl-enzyme complexes, was observed by the mass spectrometric method used here (Table 4 and Fig. 5). This is in agreement with fragmentation of the initially formed enzyme-inhibitor complexes as described by Ehmann et al. (13, 20). For all KPC variants tested, fragmentation of the avibactam complex was faster than that of the relebactam complex, with desulfated avibactam adducts more evident in spectra after 4 h of incubation and accumulating to higher levels over the duration of the experiment (Fig. 5). This low rate of relebactam desulfation is consistent with the *in crystallo* observations, with relebactam appearing to remain fully sulfated in KPC-4 crystals soaked for 1 h (Fig. 3C, Fig. S8D, E, and F). This complex shows no large differences compared with that obtained after a 16-h soak (Fig. 3F, Fig. S8A, B, and C) with the piperidine ring well defined (Fig. 3C and Fig. S8D, E, and F) and only small changes in the positions of N6, O10, and the sulfate moiety, compared with the other relebactam complexes (Fig. 4). Additional MS experiments, however, at pH values ranging from 7.0 to 8.5 revealed a significant pH dependence of desulfation, which was enhanced in a basic environment (Fig. S11). We note that in crystals soaked in acidic conditions (pH 4 to 5), no desulfation was observed for other DBOs after 3 h and, yet, did occur in longer (∼3 days), cocrystallization experiments at the same pH (16).

**TABLE 4:** Observed and calculated masses of KPC variants before and after modification due to DBO treatment

Carbapenemase by DBO treatment	Mass (Da) of:				Assignment^*d*^
	Observed^*a*^	Calculated^*b*^	Shift(s) observed^*c*^	Shift calculated	
KPC-2	30,640	30,640			
+avibactam	30,905	30,905	+265	+265	acyl
	30,826	30,825	+186	+185	acyl-80
	30,808	30,807	+168	+167	acyl-98
+relebactam	30,989	30,988	+349	+348	acyl
	30,907	30,908	+267	+268	acyl-80
	30,890	30,890	+250	+250	acyl-98
KPC-3	30,665	30,667			
+avibactam	30,931	30,932	+266	+265	acyl
	30,851	30,852	+186	+185	acyl-80
	30,834	30,834	+167	+167	acyl-98
+relebactam	31,013	31,015	+348	+348	acyl
	30,935	30,935	+270	+268	acyl-80
	30,914	30,917	+249	+250	acyl-98
KPC-4	30,657	30,657			
+avibactam	30,922	30,922	+265	+265	acyl
	30,841	30,842	+184	+185	acyl-80
	30,824	30,824	+167	+167	acyl-98
+relebactam	31,005	31,005	+348	+348	acyl
	30,924	30,925	+267	+268	acyl-80
	30,908	30,907	+251	+250	acyl-98

a Masses implied from maximum entropy deconvolution of measured spectra.

b Masses were calculated based on protein sequences without an N-terminal methionine. All differences between measured and expected masses are within experimental error.

c Corresponding to the observed protein masses.

d Acyl denotes a mass shift corresponding to reaction of an intact DBO molecule. A chemical scheme depicting the assigned acyl, acyl-80, and acyl-98 species is displayed in Fig. 5A.

These data provide clear evidence that, while relebactam:KPC acyl-enzymes can undergo limited desulfation, with the enzymes tested here, this occurs much more slowly than for avibactam. It has previously been suggested, based upon *in silico* docking of relebactam into the KPC-2 active site, followed by molecular dynamics simulations, that movements of water molecules away from the sulfate moiety (compared with their positions in avibactam complexes) increase the stability to desulfation of relebactam (21). However, even though we observe a lower rate of relebactam desulfation than avibactam, our KPC-2, -3, and -4 crystal structures (determined at higher resolution than the previous KPC-2:avibactam complex) (34) all contain an additional active site water molecule (Wat4) (Fig. 4) close to the relebactam sulfate group that is not present in the avibactam complex. Thus, the reason for the increased stability of relebactam, and the mechanism by which the nature of the R1 side chain on the DBO core structure affects the desulfation rate, remains uncertain.

### Conclusions.

Diazabicyclooctanes are an emerging and evolving class of BLIs, with the core scaffold capable of accepting modifications at the C2 position that allow further iterations to improve efficacy. Here, we demonstrate that relebactam, the most recent DBO to enter phase 3 clinical trials, inhibits the diverse, clinically relevant class A SBLs L2 and CTX-M-15 and three KPC variants, albeit at reduced potency compared with avibactam. This reduction in potency *in vitro* is not enough to impair the effectiveness of relebactam combinations against the relatively permeable K. pneumoniae Ecl8 and, yet, does impact efficacy against other organisms. Indeed, compared with the aztreonam:avibactam combination currently being developed for clinical use, an aztreonam:relebactam combination showed decreased efficacy against S. maltophilia K279a, indicating likely limitations in the effectiveness of relebactam combinations against less permeable pathogens. This is consistent with a previous report that relebactam:imipenem combinations are ineffective against Acinetobacter baumannii (14).

Our structural data show unfavorable clashes of the relebactam piperidine ring with β-lactamase residues 104 (CTX-M-15) and 105 (L2 and KPCs) that may explain differences in potency between DBOs. Indeed, the DBO compound WCK-4234, which contains the shortest R1 side chain of those tested to date, displays the greatest potency against class A SBLs, as well as, surprisingly, inhibiting a class D enzyme, OXA-48, with a *K_i_* value of 0.29 μM, which compares favorably with values of 30 μM for avibactam and of >100 μM for relebactam (16). In addition, DBOs with modifications at the C3 and C4 positions, and yet small C2 modifications, also show promising potency across SBL classes, with several compounds, for example ETX2514, exhibiting nanomolar inhibition in IC_50_ assays (17). Our crystal structures also highlight that, compared with avibactam, relebactam makes fewer interactions in the CTX-M-15 complex, which likely contributes to a reduction in potency against this enzyme. Our observations also provide evidence that the relebactam:KPC carbamylated enzyme complex can desulfate, albeit more slowly than that formed with avibactam. These data indicate that the identity of the R1 (C2) side chain of DBOs can influence desulfation, although the underlying mechanism remains to be elucidated. As desulfation prevents recyclization of the inhibitor, leading ultimately to the release of inactive degradation products and recovery of active enzyme, this could affect the potency and longevity of the inhibitor. While the timescale of relebactam desulfation that we observe here is noticeably slower than that for avibactam, likely limiting the immediate clinical relevance of this mechanism, its existence raises the possibility that KPC variants capable of supporting faster desulfation may emerge under selection pressure imposed by DBO use. For these reasons the mechanism and determinants of DBO desulfation by different class A β-lactamases deserve more detailed investigation.

The DBO scaffold and current derivations are extremely important additions to the therapeutic arsenal against resistant Gram-negative pathogens. Nevertheless, differences between individual DBOs in potency toward specific enzymes can impact the efficacy of treating problematic β-lactamase-producing pathogens, especially “difficult” organisms, such as S. maltophilia. Our extensive comparisons highlight these differences and provide significant insights that may guide further development of the core DBO inhibitor scaffold, in particular by emphasizing the need to consider the possible impact of C2 substitution on the susceptibility of the carbamylated KPC complex to degradation as well as upon interactions with the β-lactamase active site.

## MATERIALS AND METHODS

### MIC determination.

The pUBYT vector containing *bla*_KPC-3_ under the ISK_pn7_ promoter was used as a template for site-directed mutagenesis to create pUBYT containing *bla*_KPC-2_ and *bla*_KPC-4_ with the same promoter (45). The single point mutation in KPC-2 (Y274H) and double point mutations in KPC-4 (P104R, V240G) were introduced using a QuikChange Lightning site-directed mutagenesis kit (Agilent Genomics) with the primers specified in Table S1 in the supplemental material. Klebsiella pneumoniae Ecl8 was transformed with the resulting pUBYT constructs via electroporation.

S. maltophilia K279a is a well-characterized isolate from Bristol, United Kingdom, and was obtained as previously reported (46).

MIC values were determined using broth microdilution, in triplicate, in cation-adjusted Mueller-Hinton broth (Sigma) according to the Clinical and Laboratory Standards Institute (CLSI) guidelines (47). Experiments were performed in microtiter plates (Corning) containing medium with ceftazidime, imipenem, or aztreonam with inhibitor (4 mg liter^−1^ avibactam [MedChemExpress] or relebactam [MedChemExpress] dissolved in dimethyl sulfoxide). Plates were incubated overnight at 37°C for 18 to 24 h, and the absorbance at 600 nm was read using a POLARstar Omega (BMG LabTech) plate reader.

### Protein purification and crystallization.

The L2 β-lactamase was purified and crystallized as described previously (25). The mature polypeptide (codons 28 to 290) of CTX-M-15 in the expression vector pOPINF (48) was expressed in SoluBL21 (DE3) E. coli cells (Genlantis) and grown in 2xYT medium supplemented with 50 μg/ml carbenicillin to produce N-terminally His-tagged CTX-M-15. Three liters of culture was incubated at 37°C until reaching an optical density at 600 nm (OD_600_) of 0.8 and subsequently grown at 18°C overnight with 0.75 mM IPTG to induce protein expression. Cells were harvested by centrifugation (6,500 × *g*, 10 min) and resuspended in 100 ml of 50 mM HEPES (pH 7.5) and 400 mM NaCl (buffer A) with complete EDTA-free protease inhibitor (Roche), 2 μl benzonase endonuclease, and lysozyme (Sigma). Homogenized cells were lysed with 2 passages through a cell disruptor (25 kpsi) and pelleted at 100,000 × *g* for 1 h. Following the addition of 10 mM imidazole, the supernatant was incubated with 4 ml of Ni-NTA resin (Qiagen) for 1.5 h. Protein-bound resin was washed in 80 ml of buffer A plus 10 mM imidazole followed by 40 ml of buffer A plus 20 mM imidazole. Protein was eluted with buffer A plus 400 mM imidazole and concentrated in an Amicon 10-kDa molecular weight cutoff (MWCO) centrifugal filter. The imidazole concentration was reduced to 10 mM before the addition of 3C protease overnight at 4°C to remove the N-terminal His-tag. Cleaved tags were captured on Ni-NTA resin following incubation for 1 h. CTX-M-15 was loaded onto a Superdex S75 column (GE Healthcare) equilibrated with 50 mM HEPES (pH 7.5) and 150 mM NaCl and peak fractions analyzed by SDS-PAGE. Fractions assessed as >95% pure were pooled and concentrated to 37 mg ml^−1^ using an Amicon 10-kDa MWCO centrifugal filter. CTX-M-15 was crystallized using sitting-drop vapor diffusion in CrysChem 24-well plates (Hampton Research) at 20°C based on a method previously described (19). Drops comprised 1 μl of protein (15 to 37 mg/ml) and 1 μl of crystallization reagent (0.1 M Tris [pH 8.0] and 2.4 M ammonium sulfate) and were equilibrated against 500-μl reagent.

For the KPC variants (KPC-2, KPC-3, and KPC-4) codons 25 to 293 were cloned into pET28a (Noavagen) and expressed in E. coli BL21(DE3) (Novagen). Cells harboring the KPC expression vectors were grown in auto induction medium (Formedium) supplemented with 50 μg/ml kanamycin at 37°C for 8 hours and then at 18°C for 16 hours. Cells were harvested by centrifugation (6,500 × *g*, 10 min) and then resuspended in 40 ml of 20 mM Tris (pH 8.0) and 300 mM NaCl (buffer B) with a complete EDTA-free protease inhibitor (Roche), 2 μl benzonase endonuclease, and lysozyme. Homogenized cells were lysed with 2 passages through a cell disruptor (25 kpsi) and then pelleted (100,000 × *g*, 1 h). Following the addition of 10 mM imidazole, the supernatant was loaded on to a 5-ml His-trap column (GE Healthcare) equilibrated with buffer B. The His-tagged protein was eluted by a linear imidazole gradient (20 to 300 mM), and fractions were analyzed by SDS-PAGE. Fractions containing KPC were pooled and loaded onto a Superdex S75 column equilibrated with buffer B, and peak fractions were analyzed by SDS-PAGE. Fractions assessed as >95% pure were pooled and concentrated to 16.3 mg ml^−1^ KPC-2, 18.2 mg ml^−1^ KPC-3, and 14.5 mg ml^−1^ KPC-4 ^by^ using an Amicon 10-kDa MWCO centrifugal filter.

KPC-2 was crystallized using sitting-drop vapor diffusion in CrysChem 24-well plates (Hampton Research) at 20°C based upon previously described conditions (40). Drops comprised 2 μl of protein (16.3 mg ml^−1^) and 1 μl of crystallization reagent (2.0 M ammonium sulfate and 5% vol/vol ethanol) and were equilibrated against 500 μl of reagent. Initial crystals were optimized by seeding with a Seed Bead kit (Hampton Research). Drops comprised 2 μl of protein (16.3 mg ml^−1^), 1 μl of crystal seed, and 1 μl of crystallization reagent and were equilibrated against 500 μl of reagent. KPC-3 and KPC-4 crystals were grown using the same conditions, using the KPC-2 crystal seed.

### Inhibitor soaking, data collection, and structure determination.

Crystals of L2, CTX-M-15, KPC-2, KPC-3, and KPC-4 were soaked in mother liquor supplemented with 1 mM relebactam. Crystals were then briefly exposed to mother liquor containing 30% glycerol and flash-frozen in liquid nitrogen. Diffraction data for native and inhibitor-soaked crystals were collected at Diamond Light Source on beamlines I03 (L2 and CTX-M), I04 (KPC-3 and KPC-4), and I24 (KPC-2). Images were indexed and integrated using Dials (49) in the Xia2 (50) pipeline at Diamond Light Source and subsequently scaled in aimless (CCP4 suite [51]). Data were phased by molecular replacement in Phaser (52) (CCP4 suite [51]) with PDB identifiers 5NE2 (25) (L2), 4HBT (19) (CTX-M-15), and 5UL8 (40) (KPCs) as the starting structures. Initial refinement in Refmac (53) (CCP4 suite [51]) confirmed *F*_o_-*F*_c_ electron density, consistent with bound ligand, prior to further rounds of refinement in phenix.refine (54) and manual model building in WinCoot (55). Geometry restrains for relebactam were calculated using eLBOW, and omit maps were generated in Phenix (54) from the final model in the absence of the ligand. Figures were generated in PyMOL (56).

### Enzyme assays.

All enzyme assays were performed at 25°C in 10 mM HEPES (pH 7.5) and 150 mM NaCl, with nitrocefin hydrolysis followed at 486 nm (57) (Δε 486 = 20,500 M^−1^ cm^−1^) using Greiner half area 96-well plates and a Tecan Infinite 200 Pro microplate reader. Kinetic parameters were calculated and analyzed using GraphPad Prism 6. Steady-state parameters *k*_cat_ and *K*_M_ for nitrocefin hydrolysis were calculated by measuring initial rates of nitrocefin hydrolysis with L2 (1 nM), CTX-M-15 (1 nM), KPC-2 (10 nM), KPC-3 (10 nM), or KPC-4 (10 nM) and plotted against nitrocefin concentration. Steady-state values are provided in Table S2 in the supplemental material.

IC_50_ values were determined by following the initial rates of nitrocefin hydrolysis (50 μM) measured after 10-minute preincubation of inhibitor and enzyme (conditions as established by Cahill et al. [48]). Diazabicyclooctanes were dissolved in DMSO (100 mM) and diluted to the desired concentration in 10 mM HEPES (pH 7.5) and 150 mM NaCl. Reactions were initiated by the addition of nitrocefin, and initial rates were plotted against log_10_[diazabicyclooctane] and fitted to equation 1. Data were fitted to a four-parameter variable slope to obtain IC_50_ values.

(1) $$Y=\frac{100}{\left(1+10^{\left((\mathrm{LogIC}50-[I])*s\right)}\right)}$$

*Y* is the observed rate, [*I*] is inhibitor concentration, and *s* the concentration of substrate (nitrocefin).

The interaction between relebactam (*I*) and the five enzymes (*E*) was investigated using kinetic models described previously (equation 2) (13, 17, 20, 43, 58–60). For DBO inhibitors, interactions with SBLs may be described by two major pathways, involving the reversible formation of a covalent carbamylated complex (equation 2 [E-I], whose decarbamylation yields active enzyme and intact inhibitor) and fragmentation of bound inhibitor via desulfation and hydrolysis to liberate active enzyme and noninhibitory species (20) (Fig. 5).

(2) $$E+I\overset{k_{1}}{\underset{k_{-1}}{⇌}}E:I\overset{k_{2}}{\underset{k_{-2}}{⇌}}E\text{-}I\overset{k_{3}}{\to }E\text{-}I\prime \overset{k_{4}}{\to }E+P$$

The formation of the noncovalent (Michaelis) complex E:I is described by the equilibrium constant *K*, equivalent to *k*_−1_/*k*_1_ (reverse and forward rate constants, respectively). *k*_2_ is the first-order rate constant for carbamylation or the formation of E-I. *k*_−2_ is the first-order rate constant for the recyclization step (decarbamylation; reformation of E:I). The formation of covalent imine and desulfated complexes collectively described as E-I′ is described by *k*_3_ and the release of (inactive) inhibitor degradation product(s) P by *k*_4_.

Fragmentation of the carbamylated relebactam complex occurs at low levels and was only detected after a 4-h incubation of enzyme and inhibitor (Fig. 5C, E, and G). Accordingly, within the time frame of initial velocity experiments described here, equation 2 can be simplified to equation 3, as used to describe the slow-binding reversible enzyme inhibition (61).

(3) $$E+I\overset{k_{1}}{\underset{k_{-1}}{⇌}}E:I\overset{k_{2}}{\underset{k_{-2}}{⇌}}E\text{-}I$$

Where *k*_1_ and *k_−_*_1_ represent the association and dissociation rate constants for formation of the noncovalent complex described by *K*, and *k*_2_ and *k_−_*_2_ represent the carbamylation and decarbamylation (recyclization) rate constants, respectively.

The apparent inhibition constant *K_iapp_* (equation 3) (16, 21, 33, 62–65) and second-order rate constant for the onset of carbamylation by relebactam *k*_2_/*K* (see also references 13, 17, 20, 43, 58–60) across all enzymes were determined through direct competition assays of relebactam and nitrocefin under steady-state conditions. Nitrocefin was used at a fixed concentration of 50 μM; enzyme concentrations used were 1 nM (L2), 2 nM (CTX-M-15), or 10 nM (KPC-2, KPC-3, and KPC-4). The uncorrected value for *K_iapp_* (*K_iapp_′*) was then determined from Dixon plots (32), of the initial rates (*v*_0_) of nitrocefin hydrolysis (μM/sec) measured in the presence of increasing concentrations of relebactam without preincubation. The reciprocals of these initial rates (1/*v*_0_) were plotted against relebactam concentration [*I*], giving a straight line for which the value of the intercept divided by the slope gives *K_iapp_′*. These data were corrected to account for the *K*_M_ for nitrocefin [*K*_M(NCF)_, as determined experimentally, data in Table S2] using equation 4 to generate values for *K_iapp_*.

(4) $$K_{\mathrm{iapp}}K_{\mathrm{iap}p\prime }/1+\left(\frac{\left[S\right]}{K_{\text{M}\left(\text{NCF}\right)}}\right)$$

where [*S*] is the concentration of nitrocefin.

The experiments monitoring nitrocefin hydrolysis in the presence of differing relebactam concentrations were also used to obtain values for *k_2_*/*K* (apparent second-order rate constant for the onset of carbamylation). Complete progress curves were fitted to equation 5 in order to obtain values for *k*_obs_ (pseudo-first-order rate constant for inactivation).

(5) $$A=v_{f}*t+\left(v_{0}-v_{f}\right)*\left(\left(1-e^{-k_{\text{obs}}*t}\right)/k_{\text{obs}}\right)+A_{0}$$

Where *A* is absorbance at 486 nm measured at time *t*, *v*_0_ and *v*_f_ are the initial and final velocities, and *A*_0_ the initial absorbance at 486 nm.

The apparent second-order rate constant *k_2_*/*K* was then obtained by plotting *k*_obs_ against [relebactam] ([*I*]) according to equation 6, with the uncorrected value for *k_2_*/*K* (*k_2_*/*K′*) then equal to the slope of the line.

(6) $$k_{\text{obs}}=k_{-2}+k_{2}/K^{\prime }*\left[I\right]$$

The value obtained for *k_2_*/*K′* was then corrected using *K*_M_ values for nitrocefin [*K*_M(NCF)_, as determined experimentally, Table S2] in equation 7 (where [*S*] is nitrocefin concentration) to yield *k*_2_/*K.* Note that, although the quality of our straight-line fits for *k*_obs_ against relebactam is good, the fact that these experiments (along with those of others [21, 33]) necessitated the use of relebactam at concentrations approaching *K_iapp_* may introduce some uncertainty into values for *k*_2_/*K*.

(7) $$k_{2}/K=k_{2}/K^{\prime }*\left(\left(\frac{\left[S\right]}{K_{\text{M}\left(\text{NCF}\right)}}\right)+1\right)$$

To determine the rate of recovery of free enzyme, *k*_off_, 1 μM enzyme was incubated with 17.5 μM relebactam in kinetics buffer (50 mM HEPES[pH 7.5] and 150 mM NaCl) for 10 min at room temperature. This mixture was serially diluted, and the reaction was then assayed by the addition of nitrocefin to a final concentration of 50 μM. Final enzyme concentrations were as follows: 50 nM KPC-2, 5 nM KPC-3, 50 nM KPC-4, 50 pM CTX-M-15, and 50 pM L2. Complete progress curves were collected, and the results fitted to equation 8 to obtain *k*_off_.

(8) $$A=v_{f}*t+\left(v_{0}-v_{f}\right)*\left(1-e^{-k_{\mathrm{off}}t}\right)/k_{\mathrm{off}}+A_{0}$$

Where *A* is absorbance at 486 nm measured at time *t*, *v*_0_, and *v*_f_ are the initial and final velocities, and *A*_0_ the initial absorbance at 486 nm.

### Mass spectrometry of relebactam fragmentation in KPC variants.

To investigate modifications to the KPC enzymes by avibactam and relebactam, 3 μM enzyme in 50 mM Tris-HCl (pH 7.5) (unless stated otherwise) was incubated with 6 μM avibactam or relebactam at room temperature. Mass spectra were acquired in the positive ion mode by using an integrated autosampler/solid-phase extraction (SPE) RapidFire365 system (Agilent Technologies) coupled to an Agilent 6550 Accurate Mass QTOF mass spectrometer. After the indicated time, 50 μl of sample was loaded onto a C4 SPE cartridge (Agilent Technologies), washed with buffer D (100% [vol/vol] water and 0.1% [vol/vol] formic acid) and, subsequently, eluted to the mass spectrometer in buffer E (15% [vol/vol] water, 85% [vol/vol] acetonitrile, and 0.1% [vol/vol] formic acid). The cartridge was re-equilibrated in buffer D in between samples. Data were analyzed using MassHunter Qualitative Analysis software v.7 (Agilent Technologies) using the maximum entropy deconvolution algorithm.

### Data availability.

Coordinates and structure factors have been deposited at the Protein Data Bank under the following accession numbers: 6QW7 (L2 complexed with relebactam [16-hour soak]), 6QW8 (CTX-M-15 complexed with relebactam [16-hour soak]), 6QW9 (KPC-2 complexed with relebactam [16-hour soak]), 6QWA (KPC-3 complexed with relebactam [16-hour soak]), 6QWB (KPC-4 complexed with relebactam [16-hour soak]), 6QWC (KPC-4 complexed with relebactam [1-hour soak]), 6QWD (KPC-3), and 6QWE (KPC-4).

## Supplementary Material

Supplemental file 1

## References

[1] Ambler RP, Coulson AF, Frère JM, Ghuysen JM, Joris B, Forsman M, Levesque RC, Tiraby G, Waley SG 1991 A standard numbering scheme for the class A beta-lactamases. Biochem J 276:269–270. doi:10.1042/bj2760269. 2039479 PMC1151176

[2] Bush K, Jacoby GA 2010 Updated functional classification of beta-lactamases. Antimicrob Agents Chemother 54:969–976. doi:10.1128/AAC.01009-09. 19995920 PMC2825993

[3] Cantón R, González-Alba JM, Galán JC 2012 CTX-M enzymes: origin and diffusion. Front Microbiol 3:110. doi:10.3389/fmicb.2012.00110. 22485109 PMC3316993

[4] Arnold RS, Thom KA, Sharma S, Phillips M, Kristie Johnson J, Morgan DJ 2011 Emergence of Klebsiella pneumoniae carbapenemase-producing bacteria. South Med J 104:40–45. doi:10.1097/SMJ.0b013e3181fd7d5a. 21119555 PMC3075864

[5] Calvopiña K, Umland KD, Rydzik AM, Hinchliffe P, Brem J, Spencer J, Schofield CJ, Avison MB 2016 Sideromimic modification of lactivicin dramatically increases potency against extensively drug-resistant Stenotrophomonas maltophilia clinical isolates. Antimicrob Agents Chemother 60:4170–4175. doi:10.1128/AAC.00371-16. 27139464 PMC4914698

[6] Abbott IJ, Peleg AY 2015 Stenotrophomonas, Achromobacter, and nonmelioid Burkholderia species: antimicrobial resistance and therapeutic strategies. Semin Respir Crit Care Med 36:99–110. doi:10.1055/s-0034-1396929. 25643274

[7] Mehta SC, Rice K, Palzkill T 2015 Natural variants of the KPC-2 carbapenemase have evolved increased catalytic efficiency for ceftazidime hydrolysis at the cost of enzyme stability. PLoS Pathog 11:e1004949. doi:10.1371/journal.ppat.1004949. 26030609 PMC4452179

[8] Bonnet R 2004 Growing group of extended-spectrum beta-lactamases: the CTX-M enzymes. Antimicrob Agents Chemother 48:1–14. doi:10.1128/AAC.48.1.1-14.2004. 14693512 PMC310187

[9] Fritz RA, Alzate-Morales JH, Spencer J, Mulholland AJ, van der Kamp MW 2018 Multiscale simulations of clavulanate inhibition identify the reactive complex in class A β-lactamases and predict the efficiency of inhibition. Biochemistry 57:3560–3563. doi:10.1021/acs.biochem.8b00480. 29812917

[10] Drawz SM, Bonomo RA 2010 Three decades of beta-lactamase inhibitors. Clin Microbiol Rev 23:160–201. doi:10.1128/CMR.00037-09. 20065329 PMC2806661

[11] Papp-Wallace KM, Bonomo RA 2016 New β-lactamase inhibitors in the clinic. Infect Dis Clin North Am 30:441–464. doi:10.1016/j.idc.2016.02.007. 27208767 PMC4980828

[12] Papp-Wallace KM, Bethel CR, Distler AM, Kasuboski C, Taracila M, Bonomo RA 2010 Inhibitor resistance in the KPC-2 beta-lactamase, a preeminent property of this class A beta-lactamase. Antimicrob Agents Chemother 54:890–897. doi:10.1128/AAC.00693-09. 20008772 PMC2812178

[13] Ehmann DE, Jahić H, Ross PL, Gu RF, Hu J, Kern G, Walkup GK, Fisher SL 2012 Avibactam is a covalent, reversible, non-β-lactam β-lactamase inhibitor. Proc Natl Acad Sci U S A 109:11663–11668. doi:10.1073/pnas.1205073109. 22753474 PMC3406822

[14] Blizzard TA, Chen H, Kim S, Wu J, Bodner R, Gude C, Imbriglio J, Young K, Park YW, Ogawa A, Raghoobar S, Hairston N, Painter RE, Wisniewski D, Scapin G, Fitzgerald P, Sharma N, Lu J, Ha S, Hermes J, Hammond ML 2014 Discovery of MK-7655, a β-lactamase inhibitor for combination with Primaxin. Bioorg Med Chem Lett 24:780–785. doi:10.1016/j.bmcl.2013.12.101. 24433862

[15] Bush K, Bradford PA 2019 Interplay between β-lactamases and new β-lactamase inhibitors. Nat Rev Microbiol 17:295–306. doi:10.1038/s41579-019-0159-8. 30837684

[16] Papp-Wallace KM, Nguyen NQ, Jacobs MR, Bethel CR, Barnes MD, Kumar V, Bajaksouzian S, Rudin SD, Rather PN, Bhavsar S, Ravikumar T, Deshpande PK, Patil V, Yeole R, Bhagwat SS, Patel MV, van den Akker F, Bonomo RA 2018 Strategic approaches to overcome resistance against Gram-negative pathogens using β-lactamase inhibitors and β-lactam enhancers: activity of three novel diazabicyclooctanes WCK 5153, Zidebactam (WCK 5107), and WCK 4234. J Med Chem 61:4067–4086. doi:10.1021/acs.jmedchem.8b00091. 29627985 PMC6131718

[17] Durand-Réville TF, Guler S, Comita-Prevoir J, Chen B, Bifulco N, Huynh H, Lahiri S, Shapiro AB, McLeod SM, Carter NM, Moussa SH, Velez-Vega C, Olivier NB, McLaughlin R, Gao N, Thresher J, Palmer T, Andrews B, Giacobbe RA, Newman JV, Ehmann DE, de Jonge B, O'Donnell J, Mueller JP, Tommasi RA, Miller AA 2017 ETX2514 is a broad-spectrum β-lactamase inhibitor for the treatment of drug-resistant Gram-negative bacteria including Acinetobacter baumannii. Nat Microbiol 2:17104. doi:10.1038/nmicrobiol.2017.104. 28665414

[18] Choi H, Paton RS, Park H, Schofield CJ 2016 Investigations on recyclisation and hydrolysis in avibactam mediated serine β-lactamase inhibition. Org Biomol Chem 14:4116–4128. doi:10.1039/c6ob00353b. 27072755 PMC4847122

[19] Lahiri SD, Mangani S, Durand-Reville T, Benvenuti M, De Luca F, Sanyal G, Docquier JD 2013 Structural insight into potent broad-spectrum inhibition with reversible recyclization mechanism: avibactam in complex with CTX-M-15 and Pseudomonas aeruginosa AmpC β-lactamases. Antimicrob Agents Chemother 57:2496–2505. doi:10.1128/AAC.02247-12. 23439634 PMC3716117

[20] Ehmann DE, Jahic H, Ross PL, Gu RF, Hu J, Durand-Réville TF, Lahiri S, Thresher J, Livchak S, Gao N, Palmer T, Walkup GK, Fisher SL 2013 Kinetics of avibactam inhibition against class A, C, and D β-lactamases. J Biol Chem 288:27960–27971. doi:10.1074/jbc.M113.485979. 23913691 PMC3784710

[21] Papp-Wallace KM, Barnes MD, Alsop J, Taracila MA, Bethel CR, Becka SA, van Duin D, Kreiswirth BN, Kaye KS, Bonomo RA 2018 Relebactam is a potent inhibitor of the KPC-2 β-lactamase and restores imipenem susceptibility in KPC-producing Enterobacteriaceae. Antimicrob Agents Chemother 62:e00174-18. doi:10.1128/AAC.00174-18. 29610205 PMC5971601

[22] Zhanel GG, Lawrence CK, Adam H, Schweizer F, Zelenitsky S, Zhanel M, Lagacé-Wiens PRS, Walkty A, Denisuik A, Golden A, Gin AS, Hoban DJ, Lynch JP, Karlowsky JA 2018 Imipenem-relebactam and meropenem-vaborbactam: two novel carbapenem-β-lactamase inhibitor combinations. Drugs 78:65–98. doi:10.1007/s40265-017-0851-9. 29230684

[23] Hemarajata P, Humphries RM 2019 Ceftazidime/avibactam resistance associated with L169P mutation in the omega loop of KPC-2. J Antimicrob Chemother, in press. doi:10.1093/jac/dkz026. 30753572

[24] Papp-Wallace KM, Winkler ML, Taracila MA, Bonomo RA 2015 Variants of β-lactamase KPC-2 that are resistant to inhibition by avibactam. Antimicrob Agents Chemother 59:3710–3717. doi:10.1128/AAC.04406-14. 25666153 PMC4468660

[25] Calvopiña K, Hinchliffe P, Brem J, Heesom KJ, Johnson S, Cain R, Lohans CT, Fishwick CWG, Schofield CJ, Spencer J, Avison MB 2017 Structural/mechanistic insights into the efficacy of nonclassical β-lactamase inhibitors against extensively drug resistant Stenotrophomonas maltophilia clinical isolates. Mol Microbiol 106:492–504. doi:10.1111/mmi.13831. 28876489

[26] Levitt PS, Papp-Wallace KM, Taracila MA, Hujer AM, Winkler ML, Smith KM, Xu Y, Harris ME, Bonomo RA 2012 Exploring the role of a conserved class A residue in the Ω-loop of KPC-2 β-lactamase: a mechanism for ceftazidime hydrolysis. J Biol Chem 287:31783–31793. doi:10.1074/jbc.M112.348540. 22843686 PMC3442512

[27] Haidar G, Clancy CJ, Chen L, Samanta P, Shields RK, Kreiswirth BN, Nguyen MH 2017 Identifying spectra of activity and therapeutic niches for ceftazidime-avibactam and imipenem-relebactam against carbapenem-resistant Enterobacteriaceae. Antimicrob Agents Chemother 61:e00642-17. doi:10.1128/AAC.00642-17. 28630202 PMC5571343

[28] Shields RK, Clancy CJ, Hao B, Chen L, Press EG, Iovine NM, Kreiswirth BN, Nguyen MH 2015 Effects of Klebsiella pneumoniae carbapenemase subtypes, extended-spectrum beta-lactamases, and porin mutations on the *in vitro* activity of ceftazidime-avibactam against carbapenem-resistant K. pneumoniae. Antimicrob Agents Chemother 59:5793–5797. doi:10.1128/AAC.00548-15. 26169413 PMC4538547

[29] Forage RG, Lin EC 1982 DHA system mediating aerobic and anaerobic dissimilation of glycerol in Klebsiella pneumoniae NCIB 418. J Bacteriol 151:591–599. 6284704 10.1128/jb.151.2.591-599.1982 PMC220299

[30] Mojica MF, Papp-Wallace KM, Taracila MA, Barnes MD, Rutter JD, Jacobs MR, LiPuma JJ, Walsh TJ, Vila AJ, Bonomo RA 2017 Avibactam restores the susceptibility of clinical isolates of Stenotrophomonas maltophilia to aztreonam. Antimicrob Agents Chemother 61:e00777-17. doi:10.1128/AAC.00777-17. 28784669 PMC5610502

[31] Wolter DJ, Kurpiel PM, Woodford N, Palepou MF, Goering RV, Hanson ND 2009 Phenotypic and enzymatic comparative analysis of the novel KPC variant KPC-5 and its evolutionary variants, KPC-2 and KPC-4. Antimicrob Agents Chemother 53:557–562. doi:10.1128/AAC.00734-08. 19015357 PMC2630594

[32] Dixon M 1953 The determination of enzyme inhibitor constants. Biochem J 55:170. doi:10.1042/bj0550170. 13093635 PMC1269152

[33] Barnes MD, Bethel CR, Alsop J, Becka SA, Rutter JD, Papp-Wallace KM, Bonomo RA 2018 Inactivation of the Pseudomonas-derived cephalosporinase-3 (PDC-3) by relebactam. Antimicrob Agents Chemother 62:e02406-17. doi:10.1128/AAC.02406-17. 29530851 PMC5923161

[34] Krishnan NP, Nguyen NQ, Papp-Wallace KM, Bonomo RA, van den Akker F 2015 Inhibition of Klebsiella β-lactamases (SHV-1 and KPC-2) by avibactam: a structural study. PLoS One 10:e0136813. doi:10.1371/journal.pone.0136813. 26340563 PMC4560403

[35] Majiduddin FK, Palzkill T 2005 Amino acid residues that contribute to substrate specificity of class A beta-lactamase SME-1. Antimicrob Agents Chemother 49:3421–3427. doi:10.1128/AAC.49.8.3421-3427.2005. 16048956 PMC1196253

[36] Stewart NK, Smith CA, Frase H, Black DJ, Vakulenko SB 2015 Kinetic and structural requirements for carbapenemase activity in GES-type β-lactamases. Biochemistry 54:588–597. doi:10.1021/bi501052t. 25485972 PMC4303295

[37] Doucet N, De Wals PY, Pelletier JN 2004 Site-saturation mutagenesis of Tyr-105 reveals its importance in substrate stabilization and discrimination in TEM-1 beta-lactamase. J Biol Chem 279:46295–46303. doi:10.1074/jbc.M407606200. 15326193

[38] Doucet N, Pelletier JN 2007 Simulated annealing exploration of an active-site tyrosine in TEM-1 beta-lactamase suggests the existence of alternate conformations. Proteins 69:340–348. doi:10.1002/prot.21485. 17600829

[39] Papp-Wallace KM, Taracila M, Wallace CJ, Hujer KM, Bethel CR, Hornick JM, Bonomo RA 2010 Elucidating the role of Trp105 in the KPC-2 β-lactamase. Protein Sci 19:1714–1727. doi:10.1002/pro.454. 20662006 PMC2975135

[40] Pemberton OA, Zhang X, Chen Y 2017 Molecular basis of substrate recognition and product release by the Klebsiella pneumoniae carbapenemase (KPC-2). J Med Chem 60:3525–3530. doi:10.1021/acs.jmedchem.7b00158. 28388065 PMC5506774

[41] Petrella S, Ziental-Gelus N, Mayer C, Renard M, Jarlier V, Sougakoff W 2008 Genetic and structural insights into the dissemination potential of the extremely broad-spectrum class A beta-lactamase KPC-2 identified in an Escherichia coli strain and an Enterobacter cloacae strain isolated from the same patient in France. Antimicrob Agents Chemother 52:3725–3736. doi:10.1128/AAC.00163-08. 18625772 PMC2565876

[42] Ke W, Bethel CR, Thomson JM, Bonomo RA, van den Akker F 2007 Crystal structure of KPC-2: insights into carbapenemase activity in class A beta-lactamases. Biochemistry 46:5732–5740. doi:10.1021/bi700300u. 17441734 PMC2596071

[43] King DT, King AM, Lal SM, Wright GD, Strynadka NC 2015 Molecular mechanism of avibactam-mediated β-lactamase inhibition. ACS Infect Dis 1:175–184. doi:10.1021/acsinfecdis.5b00007. 27622530

[44] Ourghanlian C, Soroka D, Arthur M 2017 Inhibition by avibactam and clavulanate of the beta-lactamases KPC-2 and CTX-M-15 harboring the substitution N(132)G in the conserved SDN motif. Antimicrob Agents Chemother 61:e02510-16. doi:10.1128/AAC.02510-16. 28069651 PMC5328567

[45] Takebayashi Y, Wan Nur Ismah WAK, Findlay J, Heesom KJ, Zhang J, Williams OM, MacGowan AP, Avison MB 2017 Prediction of cephalosporin and carbapenem susceptibility in multi-drug resistant gram-negative bacteria using liquid chromatography-tandem mass spectrometry. bioRxiv doi:10.1101/138594.

[46] Gould VC, Okazaki A, Avison MB 2006 Beta-lactam resistance and beta-lactamase expression in clinical Stenotrophomonas maltophilia isolates having defined phylogenetic relationships. J Antimicrob Chemother 57:199–203. doi:10.1093/jac/dki453. 16352734

[47] CLSI. 2015 M100‐S25 performance standards for antimicrobial susceptibility testing; 25th informational supplement. CLSI, Wayne, PA.

[48] Cahill ST, Cain R, Wang DY, Lohans CT, Wareham DW, Oswin HP, Mohammed J, Spencer J, Fishwick CW, McDonough MA, Schofield CJ, Brem J 2017 Cyclic boronates inhibit all classes of β-lactamases. Antimicrob Agents Chemother 61:e02260-16. doi:10.1128/AAC.02260-16. 28115348 PMC5365654

[49] Winter G, Waterman DG, Parkhurst JM, Brewster AS, Gildea RJ, Gerstel M, Fuentes-Montero L, Vollmar M, Michels-Clark T, Young ID, Sauter NK, Evans G 2018 DIALS: implementation and evaluation of a new integration package. Acta Crystallogr D Struct Biol 74:85–97. doi:10.1107/S2059798317017235. 29533234 PMC5947772

[50] Winter G, Lobley CM, Prince SM 2013 Decision making in xia2. Acta Crystallogr D Biol Crystallogr 69:1260–1273. doi:10.1107/S0907444913015308. 23793152 PMC3689529

[51] Collaborative Computational Project Number 4. 1994 The CCP4 suite: programs for protein crystallography. Acta Crystallogr D Biol Crystallogr 50:760–763. doi:10.1107/S0907444994003112. 15299374

[52] McCoy AJ, Grosse-Kunstleve RW, Adams PD, Winn MD, Storoni LC, Read RJ 2007 Phaser crystallographic software. J Appl Crystallogr 40:658–674. doi:10.1107/S0021889807021206. 19461840 PMC2483472

[53] Murshudov GN, Vagin AA, Dodson EJ 1997 Refinement of macromolecular structures by the maximum-likelihood method. Acta Crystallogr D Biol Crystallogr 53:240–255. doi:10.1107/S0907444996012255. 15299926

[54] Adams PD, Afonine PV, Bunkoczi G, Chen VB, Davis IW, Echols N, Headd JJ, Hung LW, Kapral GJ, Grosse-Kunstleve RW, McCoy AJ, Moriarty NW, Oeffner R, Read RJ, Richardson DC, Richardson JS, Terwilliger TC, Zwart PH 2010 PHENIX: a comprehensive Python-based system for macromolecular structure solution. Acta Crystallogr D Biol Crystallogr 66:213–221. doi:10.1107/S0907444909052925. 20124702 PMC2815670

[55] Emsley P, Cowtan K 2004 Coot: model-building tools for molecular graphics. Acta Crystallogr D Biol Crystallogr 60:2126–2132. doi:10.1107/S0907444904019158. 15572765

[56] DeLano WL 2002 The PyMOL user’s manual, p 452. DeLano Scientific, San Carlos, California, USA.

[57] O'Callaghan CH, Morris A, Kirby SM, Shingler AH 1972 Novel method for detection of beta-lactamases by using a chromogenic cephalosporin substrate. Antimicrob Agents Chemother 1:283–288. doi:10.1128/AAC.1.4.283. 4208895 PMC444209

[58] Xu H, Hazra S, Blanchard JS 2012 NXL104 irreversibly inhibits the beta-lactamase from Mycobacterium tuberculosis. Biochemistry 51:4551–4557. doi:10.1021/bi300508r. 22587688 PMC3448018

[59] Mangat CS, Vadlamani G, Holicek V, Chu M, Larmour VLC, Vocadlo DJ, Mulvey MR, Mark BL 2019 Molecular basis for the potent inhibition of the emerging carbapenemase VCC-1 by avibactam. Antimicrob Agents Chemother 63:e02112-18. doi:10.1128/AAC.02112-18. 30782990 PMC6437526

[60] Shapiro AB, Gao N, Jahic H, Carter NM, Chen A, Miller AA 2017 Reversibility of covalent, broad-spectrum serine beta-lactamase inhibition by the diazabicyclooctenone ETX2514. ACS Infect Dis 3:833–844. doi:10.1021/acsinfecdis.7b00113. 28835096

[61] Morrison JF, Walsh CT 1988 The behavior and significance of slow-binding enzyme inhibitors. Adv Enzymol Relat Areas Mol Biol 61:201–301. 3281418 10.1002/9780470123072.ch5

[62] Jin W, Wachino JI, Yamaguchi Y, Kimura K, Kumar A, Yamada M, Morinaka A, Sakamaki Y, Yonezawa M, Kurosaki H, Arakawa Y 2017 Structural insights into the TLA-3 extended-spectrum β-lactamase and its inhibition by avibactam and OP0595. Antimicrob Agents Chemother 61:e00501-17. doi:10.1128/AAC.00501-17. 28739781 PMC5610492

[63] Papp-Wallace KM, Winkler ML, Gatta JA, Taracila MA, Chilakala S, Xu Y, Johnson JK, Bonomo RA 2014 Reclaiming the efficacy of β-lactam-β-lactamase inhibitor combinations: avibactam restores the susceptibility of CMY-2-producing Escherichia coli to ceftazidime. Antimicrob Agents Chemother 58:4290–4297. doi:10.1128/AAC.02625-14. 24820081 PMC4136032

[64] Poirel L, Ortiz De La Rosa JM, Kieffer N, Dubois V, Jayol A, Nordmann P 2018 Acquisition of extended-spectrum β-lactamase GES-6 leading to resistance to ceftolozane-tazobactam combination in Pseudomonas aeruginosa. Antimicrob Agents Chemother 63:e01809-18. doi:10.1128/AAC.01809-18. 30323045 PMC6325188

[65] Ruggiero M, Papp-Wallace KM, Taracila MA, Mojica MF, Bethel CR, Rudin SD, Zeiser ET, Gutkind G, Bonomo RA, Power P 2017 Exploring the landscape of diazabicyclooctane (DBO) inhibition: avibactam inactivation of PER-2 β-lactamase. Antimicrob Agents Chemother 61:e02476-16. doi:10.1128/AAC.02476-16. 28348157 PMC5444126

