# Supplementary material for: Molecular Basis of Class A β-Lactamase Inhibition by Relebactam
Source: Antimicrob Agents Chemother. 2019 Sep 23;63(10):e00564-19. doi: 10.1128/AAC.00564-19 (PMC6761529; doi:10.1128/AAC.00564-19)
Supplement: Supplemental file 1 [file AAC.00564-19-s0001.pdf]

Supplemental information for:

Molecular Basis of Class A β-lactamase Inhibition by Relebactam

Catherine L. Tooke[1], Philip Hinchliffe[1], Pauline A. Lang[2], Adrian J. Mulholland[3], Jürgen Brem[2], Christopher J. Schofield[2], and James Spencer[1*].

[1]School of Cellular and Molecular Medicine, Biomedical Sciences Building, University of Bristol, Bristol, BS8 1TD, United Kingdom.

[2]Department of Chemistry, University of Oxford, 12 Mansfield Road, Oxford, OX1 3TA, United Kingdom.

[3]Centre for Computational Chemistry, School of Chemistry, University of Bristol, Cantock's Close, BS8 1TS, Bristol, United Kingdom.

[*]Corresponding author: Jim.Spencer@bristol.ac.uk

Tables:

Table S1. Primers for site-directed mutagenesis.

Table S2. Nitrocefin steady-state kinetic parameters.

Table S3. Crystallographic data collection and refinement statistics.

Table S4. RSCCs from PDB validation.

Table S5. RMSD changes in C_α across crystal structures.

Figures:

Figure S1. Kinetic characterisation of relebactam inhibition of CTX-M-15.

Figure S2. Kinetic characterisation of relebactam inhibition of L2.

Figure S3. Kinetic characterisation of relebactam inhibition of KPC-3.

Figure S4. Kinetic characterisation of relebactam inhibition of KPC-4.

Figure S5. Views from crystal structures of wildtype KPC-3 and KPC-4.

Figure S6. Active site interactions of L2:relebactam and CTX-M-15:relebactam enzyme complexes.

Figure S7. Active site interactions of KPC-2:relebactam and KPC-3:relebactam enzyme complexes

Figure S8. Active site interactions of KPC-4:relebactam 1 h and 16 h complexes.

Figure S9. Unbiased omit F_o-F_c electron density for residues 104 and 105 in SBL:relebactam complexes.

Figure S10. Superpositions of DBO binding to class A β-lactamases.

Figure S11. pH influence on fragmentation of covalent avibactam and relebactam adducts.

Table S1. Primers for site-directed mutagenesis.

Primer name	Sequence
KPC-3 to KPC-2 FWD	5’ CTAACAAGGATGACAAGCACAGCGAGGCCGTCATC 3’
KPC-3 to KPC-2 REV	5’ GATGACGGCCTCGCTGTGCTTGTCATCCTTGTTAG 3’
KPC-2 to KPC4(1)FWD	5’ CAAAAATGCGCTGGTTCGGTGGTCACCCATCTCG 3’
KPC-2 to KPC4(1)REV	5’ CGAGATGGGTGACCACCGAACCAGCGCATTTTTG 3’
KPC-2 to KPC4(2)FWD	5’ AAAACCGGAACCTGCGGAGGGTATGGCACGGCAAATGAC 3’
KPC-2 to KPC4(2)REV	5’ GTCATTTGCCGTGCCATACCCTCCGCAGGTTCCGGTTTTG 3’

Table S2. Nitrocefin steady-state kinetic parameters.

	Nitrocefin parameters		
	k_{cat} (s^{-1})	K_M (μM)	k_{cat}/ K_M (μM^{-1} s^{-1})
**L2**	813 *(41)*	206 *(27)*	4
**CTX-M-15**	312 *(8)*	63 *(11)*	5
**KPC-2**	613 *(9)*	18.3 *(5)*	3
**KPC-3**	88 *(8)*	36 *(6)*	2
**KPC-4**	166 *(44)*	59 *(20)*	3

Standard errors in parentheses, n = 3 calculated in GraphPad Prism.

Table S3. Crystallographic Data Collection and Refinement Statistics.

	KPC-3 native	KPC-4 native	KPC-2:relebactam	KPC-3:relebactam	KPC-4 relebactam	KPC-4 relebactam (1hr)	CTX-M-15:relebactam	L2:relebactam
**Data collection**: Space group	$P2_12_12$	$P2_12_12$	$P2_12_12$	$P2_12_12$	$P2_12_12$	$P2_12_12$	$P2_12_12_1$	$P2_12_12_1$
Molecules/ASU	1	1	1	1	1	1	1	2
**Cell dimensions**: a, b, c (Å)	60.01, 78.62, 56.01	59.93, 78.74, 56.08	59.93, 78.54, 55.84	60.13, 78.72, 55.8	60.00, 79.2, 56.00	60.19, 78.98, 55.74	44.69, 45.43, 117.75	69.95, 84.34, 94.14
α, β, γ (°)	90.0, 90.0, 90.0	90.0, 90.0, 90.0	90.0, 90.0, 90.0	90.0, 90.0, 90.0	90.0, 90.0, 90.0	90.0, 90.0, 90.0	90.0, 90.0, 90.0	90.0, 90.0, 90.0
Wavelength(s) (Å)	0.78	0.94	0.78	0.86	0.94	0.78	0.98	0.98
**Resolution (Å)**	32.88-1.20 (1.22-1.20)	45.68- 1.40 (1.42-1.40)	32.84- 1.04 (1.06-1.04)	60.13-1.06 (1.08-1.06)	47.81- 1.04 (1.06-1.04)	40.90-1.30 (1.30-1.32)	44.54-1.10 (1.12-1.10)	62.82- 1.78 (1.82-1.78)
R_{pim}	0.033 (0.405)	0.053 (0.720)	0.032 (0.548)	0.023 (0.327)	0.027 (0.345)	0.053 (0.931)	0.029 (0.145)	0.074 (0.345)
$CC_{1/2}$	0.999 (0.599)	0.998 (0.767)	0.999 (0.726)	0.999 (0.833)	0.999 (0.774)	0.996 (0.684)	0.999 (0.959)	0.993 (0.635)
I / σ(I)	10.8 (2.0)	8.5 (1.6)	10.5 (1.6)	14.0 (2.3)	12.7 (2.2)	9.8 (1.6)	19.3 (5.9)	7.8 (2.3)
Completeness (%)	100 (100)	100 (100)	100 (100)	98.9 (97.2)	99.4 (90.8)	100 (100)	99.7 (94.7)	100 (100)
Redundancy	12.7 (12.6)	12.6 (12.4)	12.7 (12.5)	13.1 (13.2)	12.1 (7.5)	12.0 (12.1)	11.9 (6.7)	12.7 (12.8)
**Refinement**								
Resolution (Å)	32.18 -1.20	45.68 - 1.40	32.84- 1.04	55.8-1.06	45.712 – 1.04	40.90-1.30	42.50- 1.10	62.82-1.78
No. reflections	83425	52930	126,715	118,930	127, 468	75,907	97,874	54,128
R_{work} / R_{free}	0.1430 / 0.1579	0.1453 / 0.1767	0.1149 / 0.1338	0.1223 / 0.1425	0.1202 / 0.1341	0.1575 / 0.1676	0.1147 / 0.1313	0.1593 / 0.2050
No. atoms								
Protein	4310	4135	4352	4228	4363	4321	4083	8246
Solvent	364	323	387	406	458	307	397	632
Ligand	-	-	80	80	80	43	86	43
B-factors								
Protein	16.73	19.84	12.78	13.61	13.24	18.58	11.83	19.33
Solvent	32.79	35.11	33.13	32.0	30.01	32.67	26.08	31.47
Ligand	-	-	13.66	13.95	16.58	18.43	19.76	34.19
R.m.s. deviations								
Bond lengths (Å)	0.007	0.007	0.011	0.011	0.012	0.008	0.014	0.010
Bond angles (°)	1.007	0.962	1.338	1.437	1.345	0.972	1.461	1.070
Ramachandran (%)								
Outliers	0	0	0	0	0	0	0.39	0
Favoured	98.9	98.5	98.5	98.9	97.7	97.7	98.1	96. 6
PDB ID	6QWD	6QWE	6QW9	6QWA	6QWB	6QWC	6QW8	6QW7

***Values in parentheses are for highest-resolution shell.**

Table S4. Relebactam Real-Space Correlation Coefficients (RSCCs) from PDB Validation.

	RSCC relebactam	RSCC imine
KPC-2:relebactam	0.99	0.98
KPC-3:relebactam	0.98	0.97
KPC-4:relebactam (1hr)	0.97	-
KPC-4:relebactam (16 hr)	0.99	0.99
L2:relebactam	0.93	-
CTX-M-15:relebactam	0.96	-

Table S5. RMSD changes in C_α across crystal structures.

Reference structure	Moving structure	RMSD (Å)	Number of atoms
Native KPC-2 (PDB: 5UL8)	KPC-3	0.099	268
	KPC-4	0.097	268
	KPC-2:relebactam	0.126	268
Native KPC-3	KPC-3:relebactam	0.127	268
Native KPC-4	KPC-4:relebactam (1hr)	0.150	268
Native KPC-4	KPC-4:relebactam (16 hr)	0.138	268
Native L2 (PDB: 5NE2)	L2:relebactam	0.142	270
Native CTX-M-15 (PDB: 4HBT)	CTX-M-15:relebactam	0.263	261

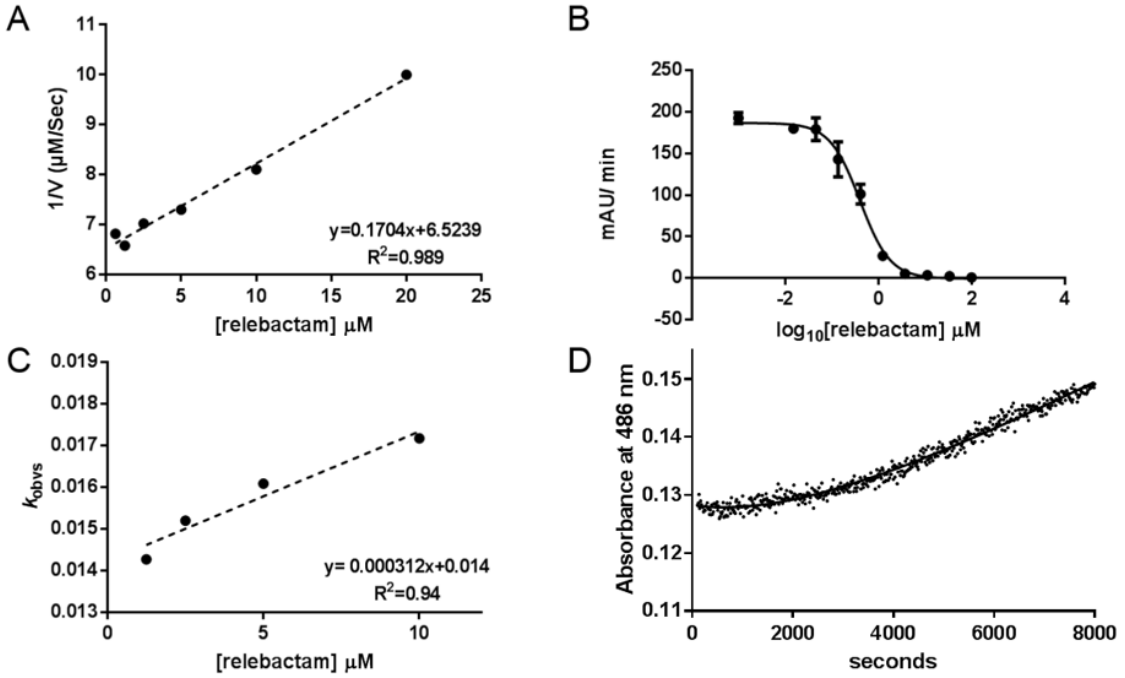

Figure S1. Kinetic Characterization of Relebactam Inhibition of CTX-M-15. (A) Dixon plot of reciprocals of initial nitrocefin hydrolysis rates (1/V) by enzyme:relebactam mixtures plotted against relebactam concentration. The apparent inhibition constant K_{iapp} is obtained from the slope of the fitted straight line. (B) Initial rates of nitrocefin hydrolysis (absorbance units/min) after 10-minute incubation with relebactam, plotted against $\log_{10}$ [relebactam]. Fitted curve is used to derive IC_{50} according to Equation 1. (C) Plot of k_{obs} (pseudo-first-order rate constant for inactivation) against relebactam concentration. The apparent second-order rate constant for the onset of carbamylation k_2/K is obtained from the slope of the fitted straight line. (D) Progress curve representing recovery of nitrocefin hydrolysis following 10 minute pre-incubation of enzyme (1 µM) with 17.5 µM relebactam, diluted to a final concentration of 50 pM enzyme. The rate of recovery of free enzyme, k_{off}, is obtained from the fitted line shown according to Equation 6.

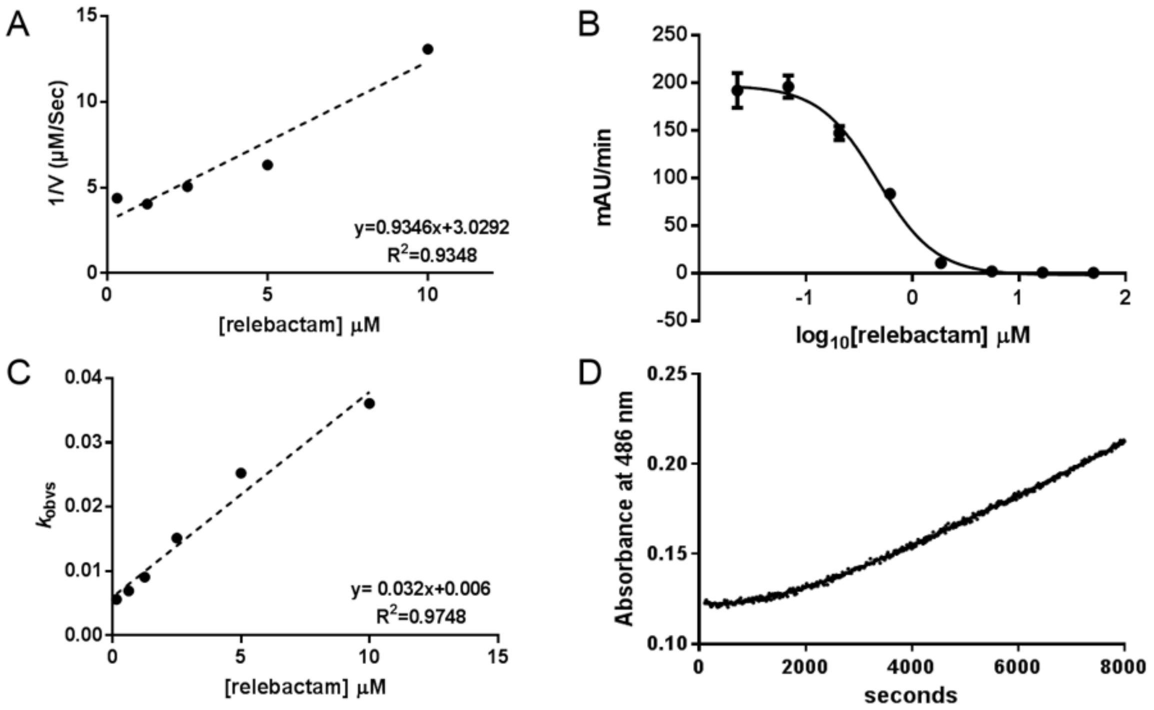

Figure S2. Kinetic Characterization of Relebactam Inhibition of L2. (A) Dixon plot of reciprocals of initial nitrocefin hydrolysis rates (1/V) by enzyme:relebactam mixtures plotted against relebactam concentration. The apparent inhibition constant K_{iapp} is obtained from the slope of the fitted straight line. (B) Initial rates of nitrocefin hydrolysis (absorbance units/min) after 10-minute incubation with relebactam, plotted against $\log_{10}$ [relebactam]. Fitted curve is used to derive IC_{50} according to Equation 1. (C) Plot of k_{obs} (pseudo-first-order rate constant for inactivation) against relebactam concentration. The apparent second-order rate constant for the onset of carbamylation k_2/K is obtained from the slope of the fitted straight line. (D) Progress curve representing recovery of nitrocefin hydrolysis following 10 minute pre-incubation of enzyme (1 µM) with 17.5 µM relebactam, diluted to a final concentration of 50 pM enzyme. The rate of recovery of free enzyme, k_{off}, is obtained from the fitted line shown according to Equation 6.

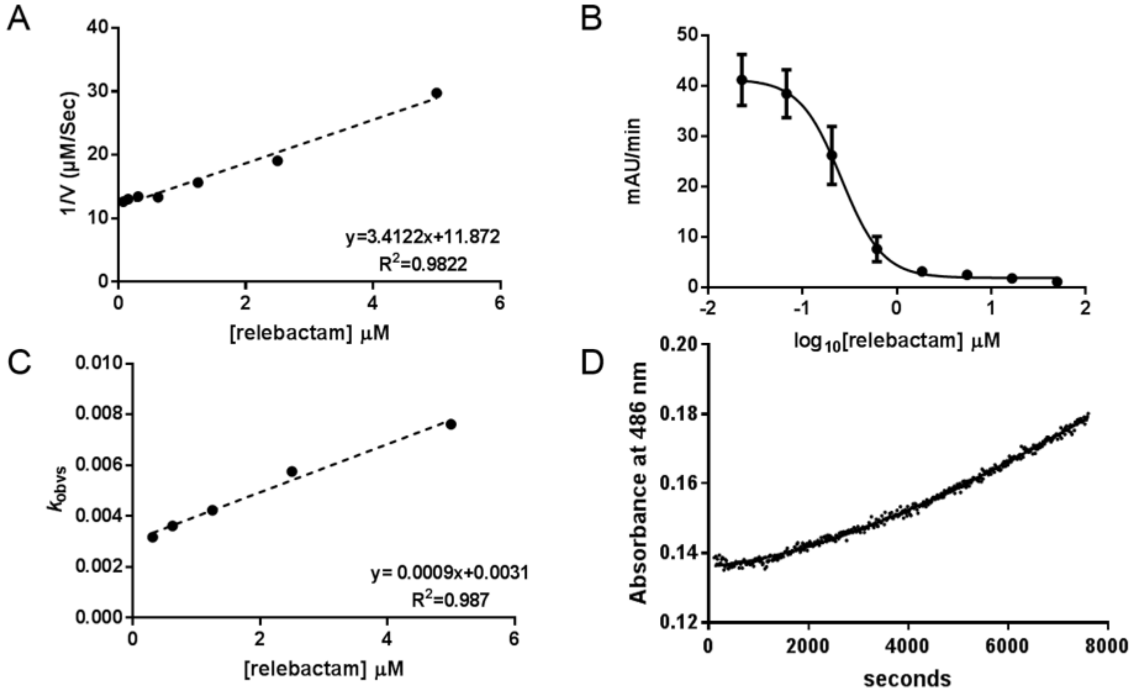

Figure S3. Kinetic Characterization of Relebactam Inhibition of KPC-3. (A) Dixon plot of reciprocals of initial nitrocefin hydrolysis rates (1/V) by enzyme:relebactam mixtures plotted against relebactam concentration. The apparent inhibition constant K_{iapp} is obtained from the slope of the fitted straight line. (B) Initial rates of nitrocefin hydrolysis (absorbance units/min) after 10-minute incubation with relebactam, plotted against $\log_{10}$ [relebactam]. Fitted curve is used to derive IC_{50} according to Equation 1. (C) Plot of k_{obs} (pseudo-first-order rate constant for inactivation) against relebactam concentration. The apparent second-order rate constant for the onset of carbamylation k_2/K is obtained from the slope of the fitted straight line. (D) Progress curve representing recovery of nitrocefin hydrolysis following 10 minute pre-incubation of enzyme (1 µM) with 17.5 µM relebactam, diluted to a final concentration of 5 nM enzyme. The rate of recovery of free enzyme, k_{off}, is obtained from the fitted line shown according to Equation 6.

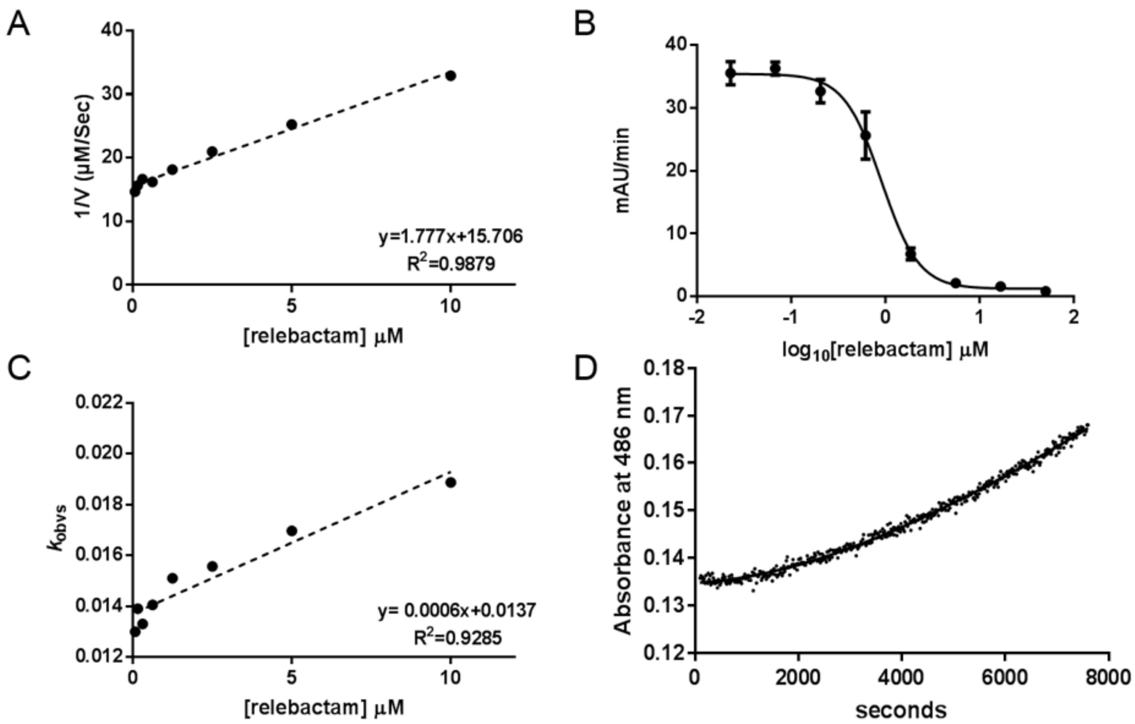

Figure S4. Kinetic characterisation of relebactam inhibition of KPC-4. (A) Dixon plot of reciprocals of initial nitrocefin hydrolysis rates (1/V) by enzyme:relebactam mixtures plotted against relebactam concentration. The apparent inhibition constant K_{iapp} is obtained from the slope of the fitted straight line. (B) Initial rates of nitrocefin hydrolysis (absorbance units/min) after 10-minute incubation with relebactam, plotted against $\log_{10}$ [relebactam]. Fitted curve is used to derive IC_{50} according to Equation 1. (C) Plot of k_{obs} (pseudo-first-order rate constant for inactivation) against relebactam concentration. The apparent second-order rate constant for the onset of carbamylation k_2/K is obtained from the slope of the fitted straight line. (D) Progress curve representing recovery of nitrocefin hydrolysis following 10 minute pre-incubation of enzyme (1 µM) with 17.5 µM relebactam, diluted to a final concentration of 50 nM enzyme. The rate of recovery of free enzyme, k_{off}, is obtained from the fitted line shown according to Equation 6.

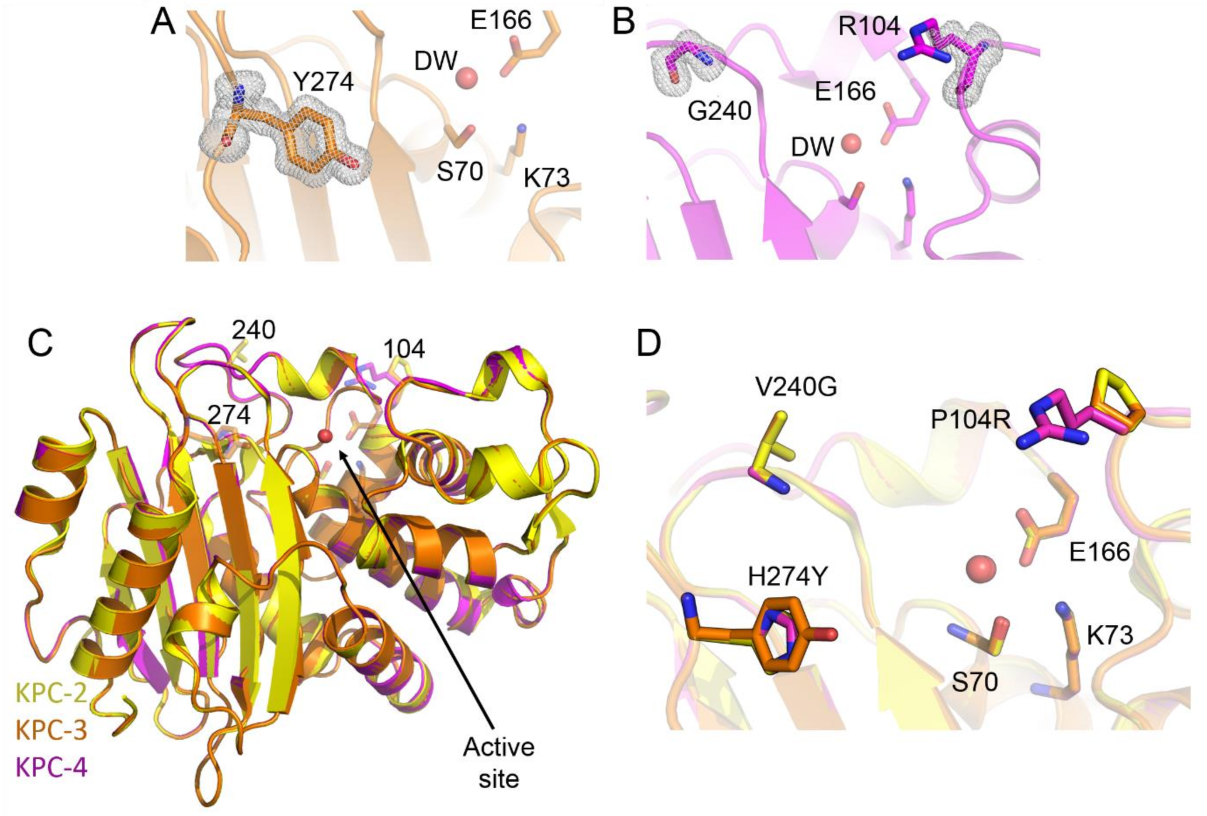

Figure S5. Views from crystal structures of wildtype KPC-3 and KPC-4. (A) Zoom in of the KPC-3 active site with unbiased omit F_o-F_c electron density for Tyr274. (B) Zoom in of the KPC-4 active site with unbiased omit F_o-F_c electron density for Gly240 and Arg104. (C) Superposition of native KPC-2 (PDB 5UL8) in yellow, KPC-3 in orange and KPC-4 in pink, variant positions are identified as sticks and labelled. (D) Close-up view of the active site in panel C. Key active site residues and variant positions shown as sticks and the 'deacylating' water (DW) represented as red spheres.

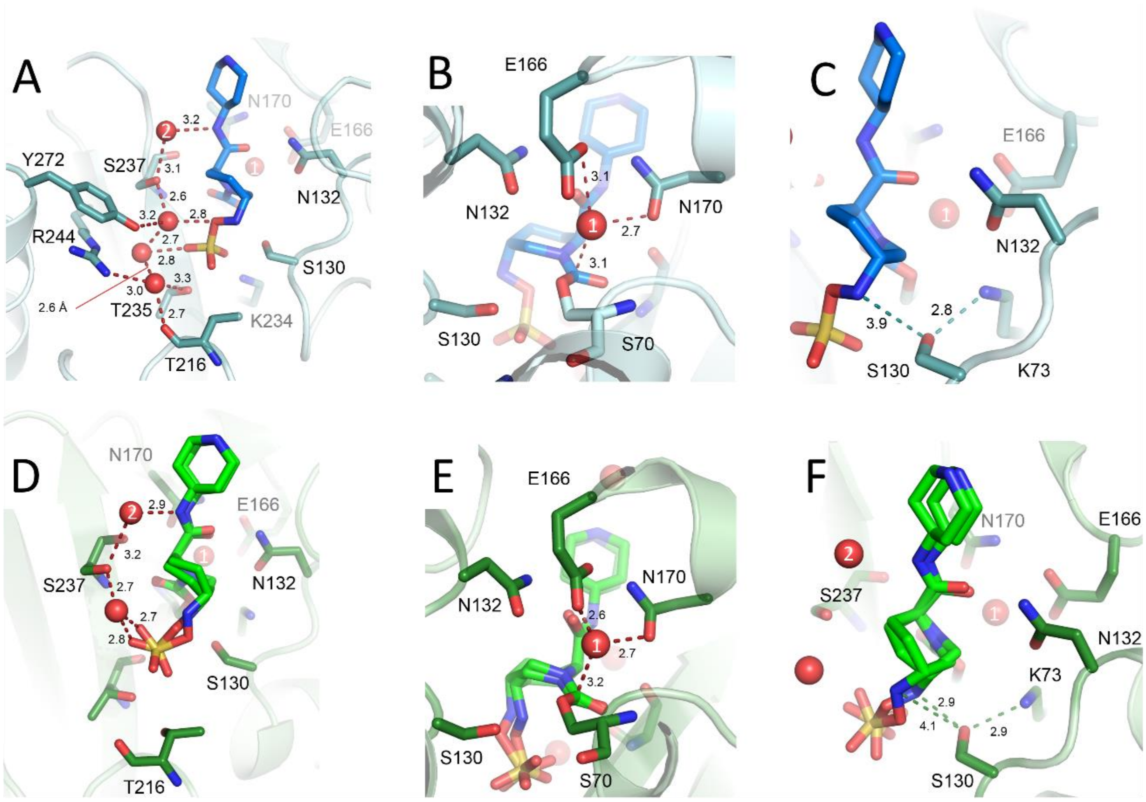

Figure S6. Active site interactions of L2:relebactam and CTX-M-15:relebactam acylenzymes. (A, B and C) L2, in teal, complexed with relebactam (blue). (D, E and F) CTX-M-15, in forest, complexed with relebactam (green). Relebactam and key active site residues represented as sticks, hydrogen bonding interactions with protein are shown as backbone colour. Waters are shown as red spheres and waters conserved in relebactam complexes across the five enzymes are numbered. Distances with waters are represented with red dashes in Å.

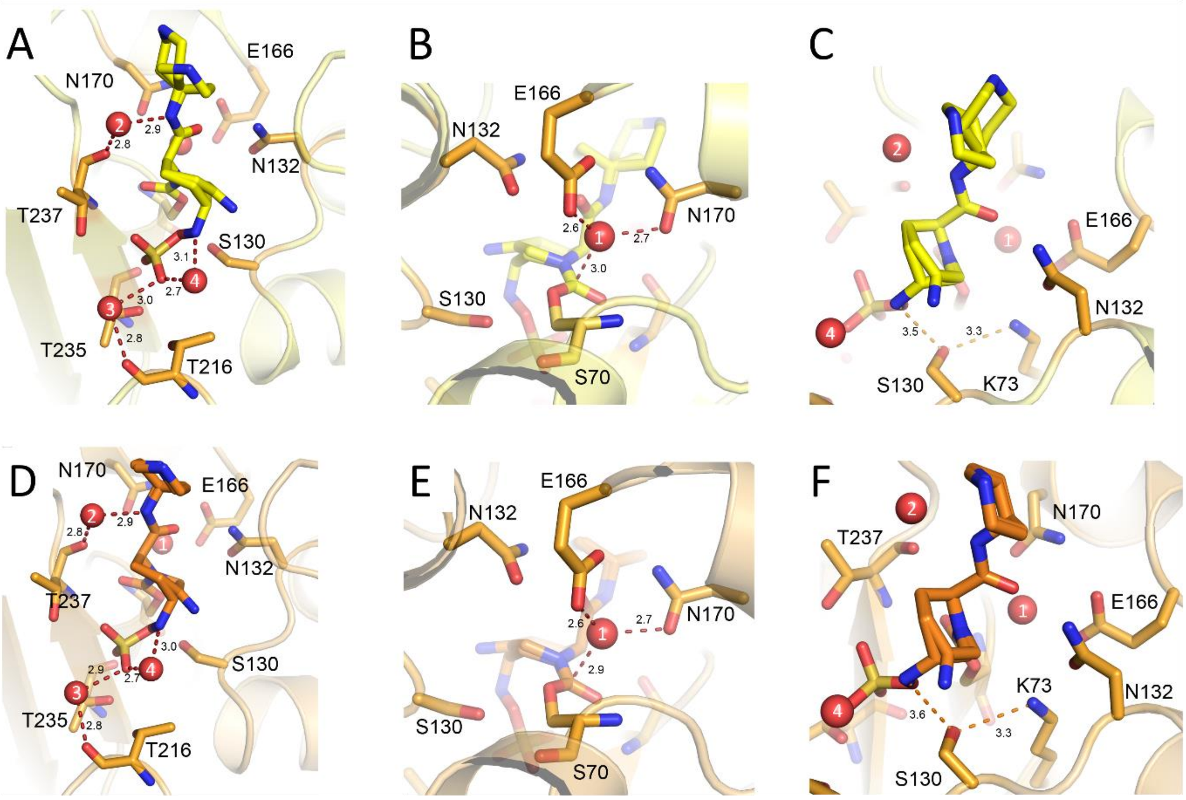

Figure S7. Active site interactions of KPC-2:relebactam and KPC-3:relebactam enzyme complexes. (A, B and C) KPC-2, light yellow, complexed with relebactam (bright yellow). (D, E and F) KPC-3, light orange, complexed with relebactam (orange). Relebactam and key active site residues represented as sticks, hydrogen bonding interactions with protein are shown as backbone colour. Waters, shown as red spheres, are numbered. Distances with waters are represented with red dashes in Å.

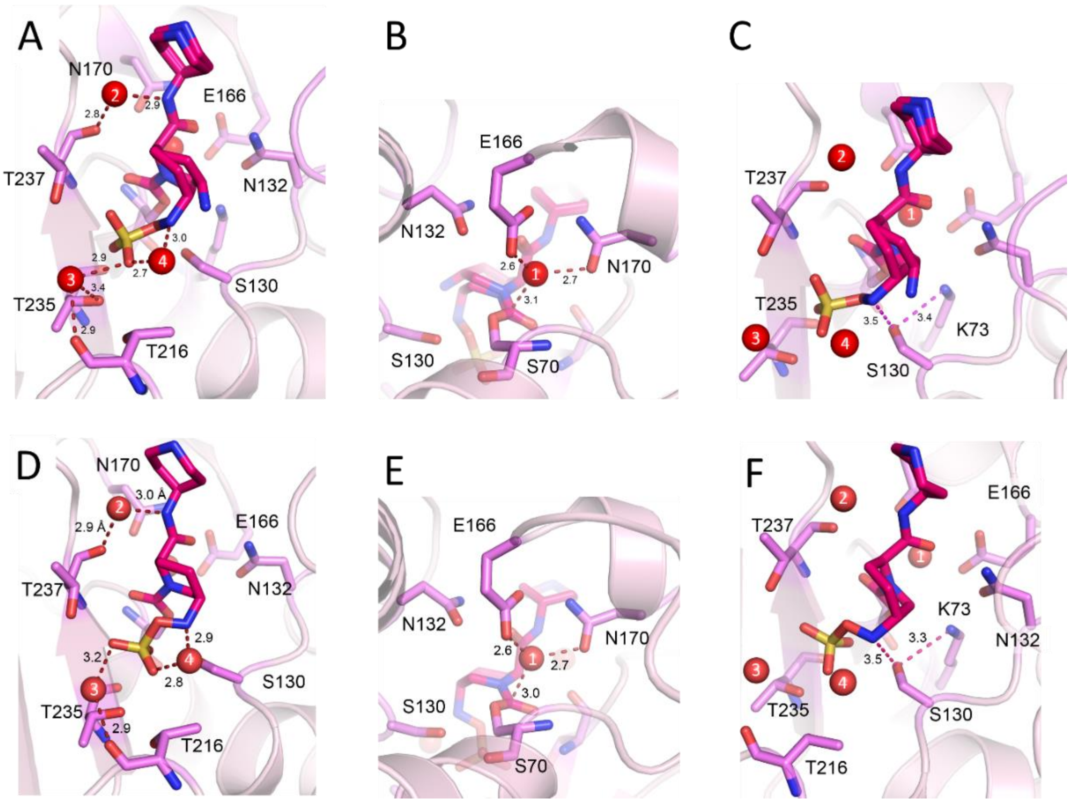

Figure S8. Active site interactions of KPC-4:relebactam 1 h and 16 h complexes. (A, B and C) KPC-4 (16-hour soak), complexed with relebactam (hot pink). (D, E and F) KPC-4 (1-hour soak), complexed with relebactam (hot pink). Relebactam and key active site residues represented as sticks, hydrogen bonding interactions with protein are shown as backbone colour. Waters, shown as red spheres, are numbered. Distances with waters are represented with red dashes in Å.

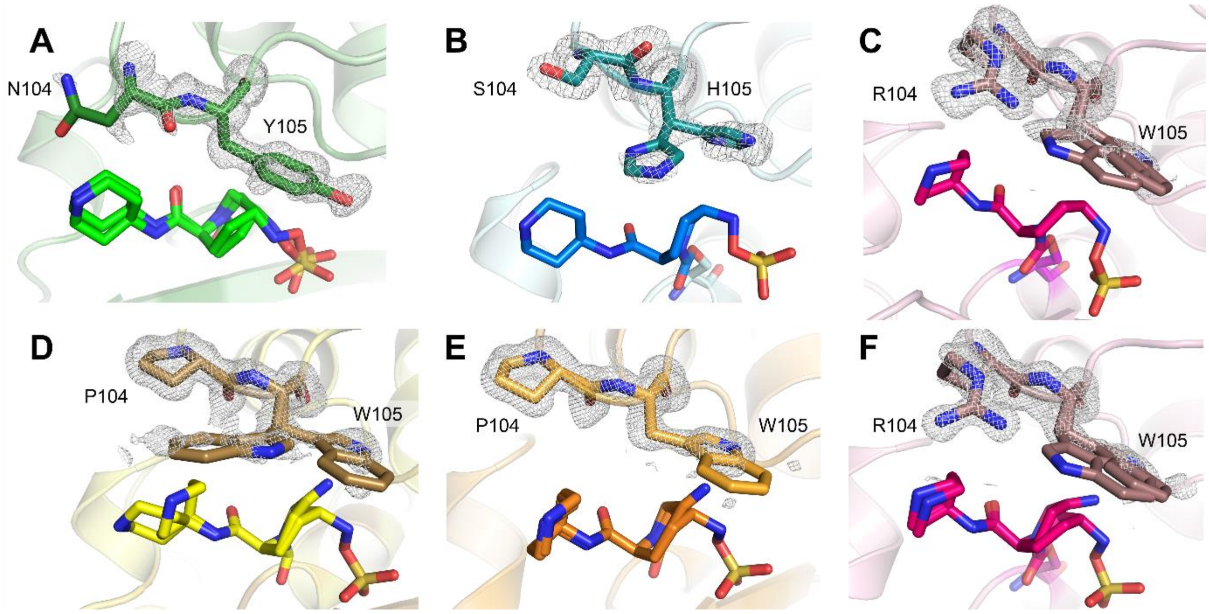

Figure S9. Unbiased omit F_o-F_c electron density for residues 104 and 105 in the SBL:relebactam complexes. (A) CTX-M-15 (green); (B) L2 (blue); (C) KPC-4 1-hour relebactam soak (pink); (D) KPC-2 (yellow); (E) KPC-3 (orange) and (F) KPC-4 16-hour relebactam soak (pink).

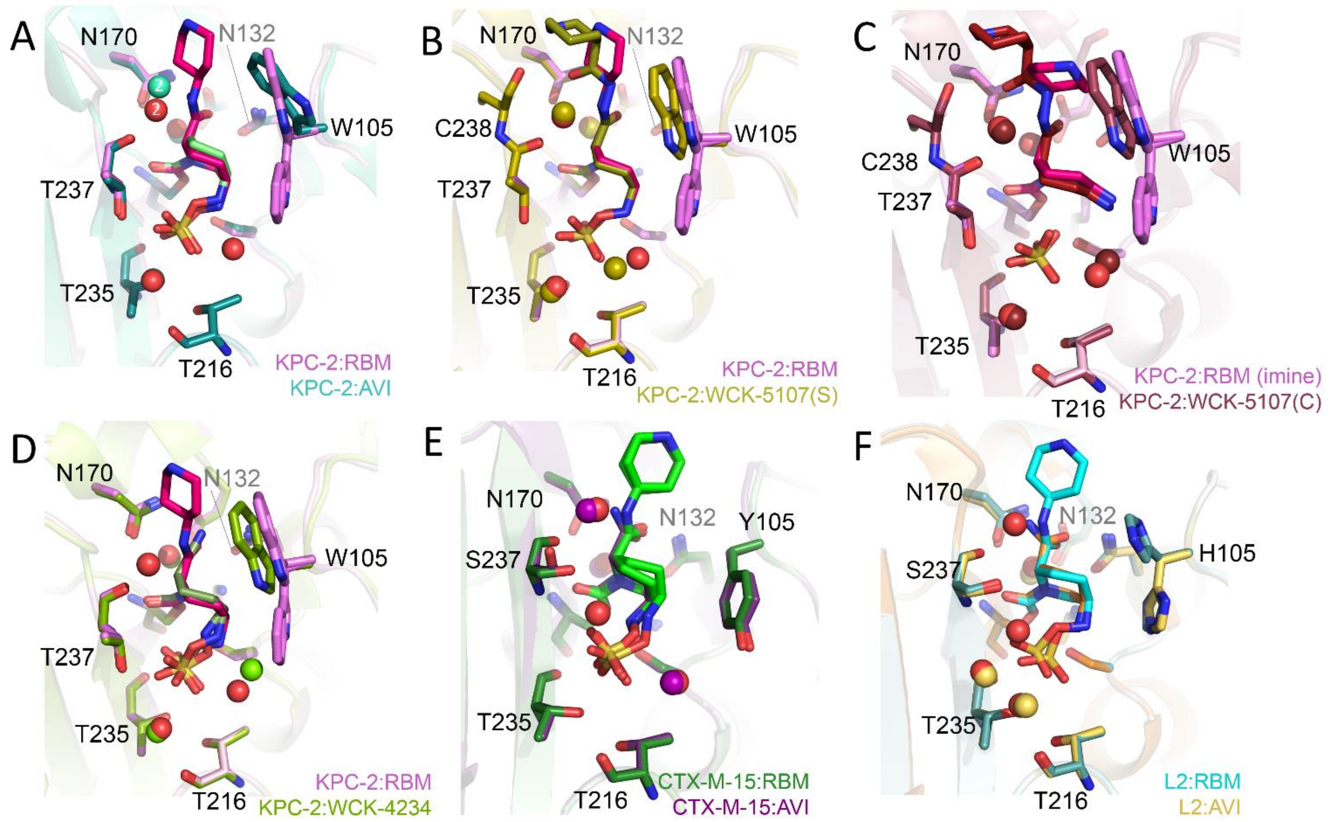

Figure S10. Superpositions of DBO binding to class A β-lactamases. (A) Avibactam (teal) overlaid with relebactam (pink) in KPC-2. (B) WCK-5107 ('intact', gold) soaked in to KPC-2 crystals (16 hours) overlaid with relebactam in KPC-2 (pink). (C) Co-crystal structure of the desulfated form of WCK-5107 (brown) superposed with the desulfated imine form of relebactam observed in KPC-2 (pink). (D) WCK-4234 (green) superposed with relebactam (pink) in KPC-2. (E) Both conformations of relebactam (green) superposed with avibactam (purple) bound in CTX-M-15. (F) Comparison of relebactam (cyan) and avibactam (yellow) binding in L2.

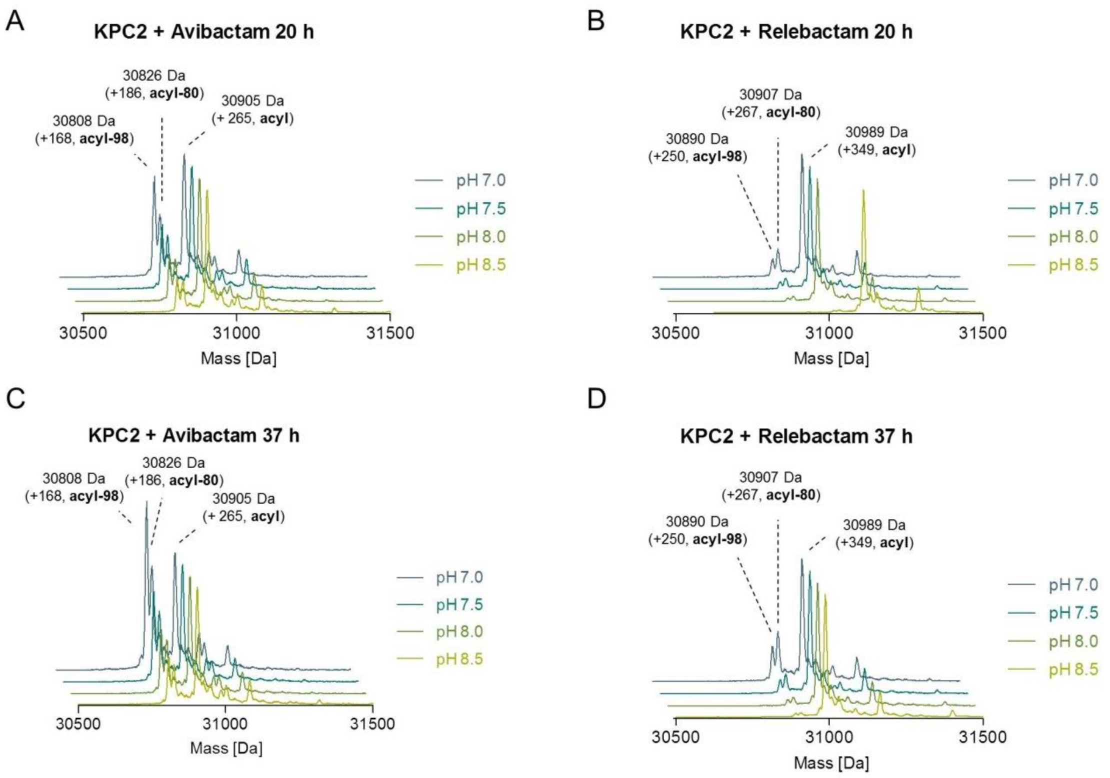

Figure S11: pH influence on fragmentation of covalent avibactam and relebactam adducts. Samples were incubated at room temperature for the indicated time in 50 mM Tris-HCl buffered to the indicated pH. The 'acyl', 'acyl' -80 (hydroxylamine) and 'acyl' -98 (imine) species are displayed in **Figure 5A**. (A) KPC-2 incubated with avibactam (20 hrs); (B) KPC-2 incubated with relebactam (20 hrs); (C) KPC-2 incubated with avibactam (37 hrs); (D) KPC-2 incubated with relebactam (37 hrs).
